# Neurological and hematological safety profiles of GSK-3β inhibitors: insights from multi-database pharmacovigilance and experimental validation

**DOI:** 10.3389/fimmu.2026.1915614

**Published:** 2026-09-17

**Authors:** Xinchi Luan, Bingcheng Fan, Xuezhe Wang, Xiaoxuan Li, Yuhui Song, Xiaolei Zhang, Huhu Zhang, Ruolan Chen, Yi Li, Zelin Yang, Ning Liu, Weiwei Qi, Wensheng Qiu, Jing Guo

**Affiliations:** 1 Department of Oncology, The Affiliated Hospital of Qingdao University, Qingdao, Shandong, China; 2 Department of Cardiology, The Affiliated Hospital of Qingdao University, Qingdao, China; 3 Massachusetts General Hospital Cancer Center, Boston, MA, United States; 4 Department of Genetics and Cell Biology, School of Basic Medicine, Qingdao University, Qingdao, China; 5 Department of Gastrointestinal Surgery, The Affiliated Hospital of Qingdao University, Qingdao, China

**Keywords:** adverse drug reactions, bibliometrics, cancer, disproportionality analysis, GSK-3β inhibitors

## Abstract

**Background:**

Glycogen synthase kinase 3 beta (GSK-3β) inhibitors have received substantial attention for their therapeutic potential; however, their systemic safety profile remains incompletely characterized.

**Objective:**

This study characterized the research landscape and exploratory safety-reporting signals associated with agents with reported GSK-3β activity by integrating bibliometric analysis, pharmacovigilance, and preliminary experimental assessment.

**Methods:**

A multi-layered framework included bibliometric analysis, disproportionality analyses of FAERS, JADER, and CVARD reports, and transcriptomic profiling. SH-SY5Y cells were treated with 9-ING-41 (1 μM, 24 h); cell viability, qRT-PCR, and DCFH-DA-based intracellular oxidative-stress measurements were assessed.

**Results:**

Bibliometric analysis showed sustained growth in GSK-3β-related research. Pharmacovigilance identified neurological and hematologic disproportionality signals across 27 System Organ Classes. In SH-SY5Y cells, 9-ING-41 was associated with modest changes in neuronal-function, inflammatory-response, and GSK-3β/Wnt-pathway transcripts, increased DCFH-DA fluorescence, and high cell viability. These cell-based observations are preliminary and do not establish clinical causality.

**Conclusion:**

The literature-derived study-drug panel showed exploratory neurological and hematologic reporting signals. Cross-database recurrence can prioritize hypotheses, whereas pharmacological heterogeneity and the limitations of spontaneous reporting require cautious interpretation. The SH-SY5Y experiments provide preliminary biological plausibility only.

## 1 Introduction

Cancer remains a leading cause of death worldwide and imposes a substantial public-health burden (1, 2). A Global Burden of Disease analysis reported marked increases in incident cancer and cancer mortality from 1990 to 2023 and projected further increases through 2050 (3). Standard treatment includes surgery, chemotherapy, radiotherapy, molecularly targeted therapy, chimeric antigen receptor T-cell therapy, and immunotherapy (4–8). Despite these advances, limited efficacy and treatment-related toxicities remain major barriers for many patients. New targets are therefore needed to improve benefit while limiting harm. Glycogen synthase kinase 3 beta (GSK-3β) is one such candidate target.

GSK-3β is a serine/threonine kinase that plays a crucial role in a wide array of cellular processes, including cell cycle regulation, apoptosis, and signal transduction (9–11). GSK-3β is involved in the regulation of various signaling pathways, most notably the Wnt/β-catenin pathway, which is pivotal for cell proliferation and differentiation (12, 13). Aberrant GSK-3β is a key driver in the pathogenesis and progression of numerous diseases, including cancer, where its dysregulation of critical signaling networks actively promotes tumorigenesis (11, 14, 15). In recent years, GSK-3β inhibitors have emerged as potential therapeutic candidates owing to their capacity to broadly modulate these pathways, either by direct enzyme inhibition or by indirect influence on associated molecular targets. These inhibitors have shown demonstrated preclinical efficacy across a spectrum of malignancies, notably breast, colorectal, and prostate cancers. Their mechanism of action is multifaceted, often involving the induction of cell cycle arrest and apoptosis, which also serves to potentiate the effectiveness of existing anticancer treatments (14, 16, 17). Despite their considerable therapeutic promise, the clinical translation of GSK-3β inhibitors in oncology faces persistent challenges, underscoring the critical need for a more comprehensive understanding of their definitive efficacy and long-term safety profiles. Notably, as GSK-3β plays a role in numerous biological functions, the use of its inhibitors could lead to unintended off-target effects (18, 19). To this end, the identification of GSK-3β inhibitors with superior safety profiles, alongside the robust prediction of potential adverse drug reactions, is critical for optimizing their clinical development in cancer therapy.

In recent years, bibliometric analysis has become an increasingly valuable approach for mapping scientific knowledge structures and identifying emerging research frontiers (20). By integrating quantitative data from published literature, bibliometrics enables researchers to visualize trends, evaluate influential contributors, and uncover evolving hotspots within a specific research field (21). Meanwhile, pharmacovigilance databases such as the U.S. Food and Drug Administration Adverse Event Reporting System (FAERS), the Japanese Adverse Drug Event Report (JADER) and the Canadian Vigilance Adverse Reaction Database (CVARD) provide large-scale, real-world evidence that can reveal potential safety signals and drug-associated adverse events.

Previous reports have generally examined bibliometric trends, spontaneous-reporting signals, or experimental mechanisms in isolation. This study integrates all three levels: (i) bibliometric mapping of GSK-3β inhibitor research in cancer; (ii) parallel, multi-level disproportionality analyses in FAERS, JADER, and CVARD; and (iii) transcriptomic and cell-based assessment of biological plausibility. The cross-database design permits differentiation of signals observed in more than one database from database-specific reporting patterns, while the experimental component is explicitly interpreted as preliminary mechanistic support rather than evidence of clinical causality.

The study aimed to characterize the research landscape and exploratory safety signals of a literature-derived panel of agents with reported GSK-3β activity in cancer-related research. Bibliometric mapping was used to describe temporal trends, influential contributors, and therapeutic contexts. The pharmacovigilance component assessed disproportionate reporting of adverse events, not incidence or causal risk, and the experimental component evaluated selected neurobiological responses to 9-ING-41 (20–22).

## 2 Methods

### 2.1 Overview

This study consists of three complementary components: bibliometric analysis, pharmacovigilance analysis, and experimental validation, which together provide a comprehensive evaluation of the research landscape and safety profiles of GSK-3β inhibitors.

The first component employs bibliometric methods to systematically characterize the development of the GSK-3β inhibitor research field from multiple perspectives. The analysis evaluates the contributions and collaborative relationships of authors, institutions, and countries to identify major research forces and cooperation networks. Publication trends are evaluated to assess the evolution and growth of the field over time. Journal analysis is performed to identify influential journals based on publication output and citation impact. Citation analysis is further conducted to identify highly cited studies and reveal the intellectual foundation of the field. In addition, keyword co-occurrence and clustering analyses are used to identify research hotspots, emerging topics, and potential future directions. Collectively, these analyses provide a comprehensive overview of the current status and developmental trajectory of GSK-3β inhibitor research.

The second component focuses on the pharmacovigilance assessment of GSK-3β inhibitors identified through literature retrieval. AE data were collected from three major pharmacovigilance databases, including the Food and Drug Administration Adverse Event Reporting System (FAERS), the Japanese Adverse Drug Event Report (JADER) database, and the Canadian Vigilance Adverse Reaction Database (CVARD). AE signals were systematically evaluated across multiple MedDRA hierarchical levels, including System Organ Class (SOC), High Level Group Term (HLGT), High Level Term (HLT), and Preferred Term (PT). Clinical prioritization analyses were performed to assess the potential clinical significance of identified signals. Furthermore, time-to-onset (TTO) analyses were conducted to characterize the temporal patterns of AE occurrence following drug administration. Subgroup analyses stratified by sex and age were also performed to investigate potential demographic differences in AE reporting.

The third component was designed to provide preliminary experimental support for the biological plausibility of selected pharmacovigilance and transcriptomic observations. Human neuroblastoma SH-SY5Y cells were treated with the GSK-3β inhibitor 9-ING-41. Cell viability was assessed using the CCK-8 assay to select a concentration compatible with molecular measurements. Quantitative real-time PCR was used to evaluate genes related to neuronal function, inflammatory responses, and GSK-3β/Wnt signaling, and intracellular reactive oxygen species (ROS) were measured as an index of oxidative stress. These experiments do not establish clinical causality; instead, they identify cell-based changes that may inform hypotheses for future mechanistic and clinical studies.

An overview of the overall study design and analytical workflow is presented in **Figure 1**.

**Figure 1:**
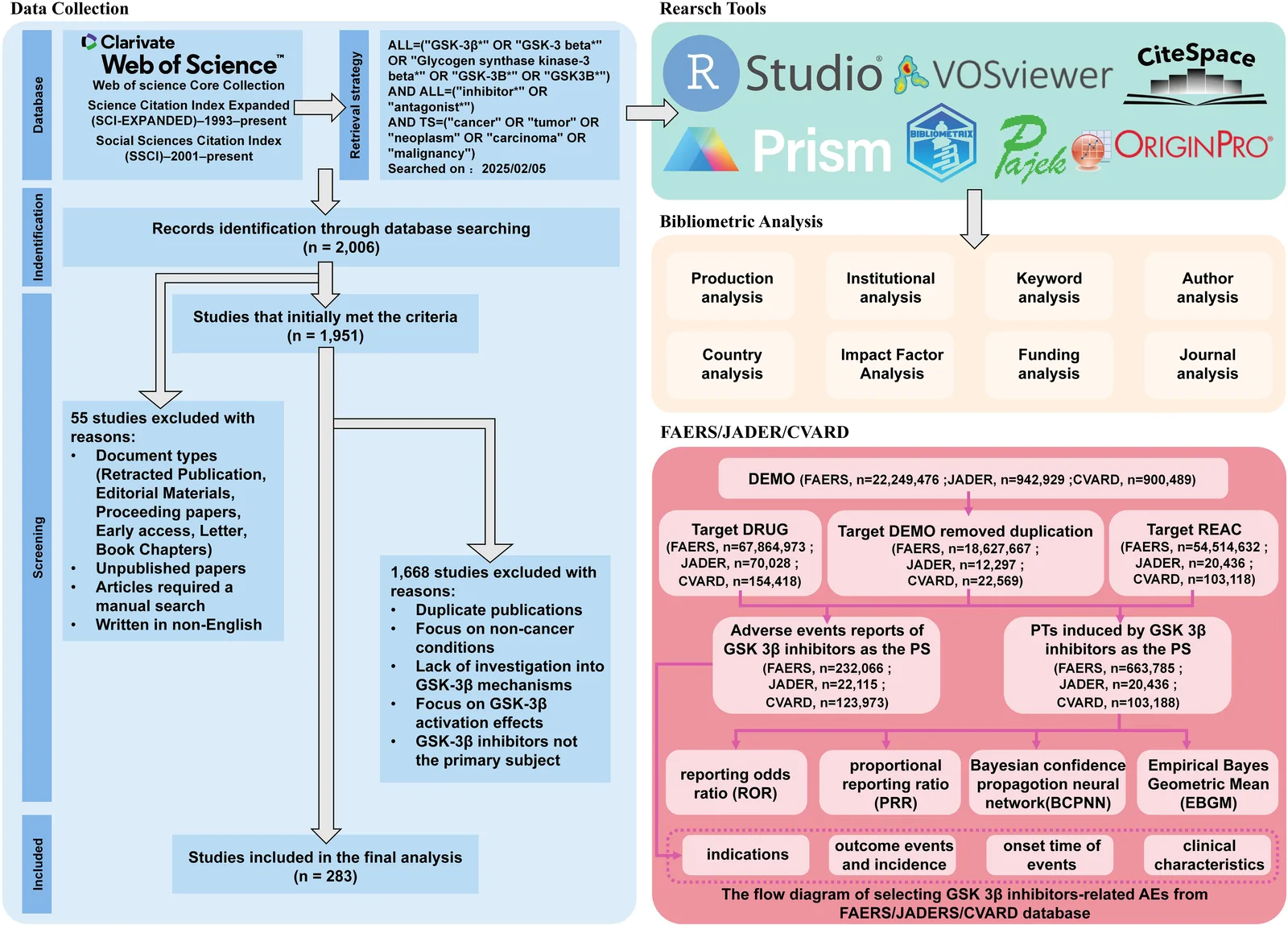
Overview of the study framework. This study is divided into two main sections: bibliometric analysis and adverse drug reaction (ADR) analysis. The Bibliometric Analysis investigates the research landscape through the examination of publication trends, researcher, institution, and country contributions, as well as interdisciplinary collaboration patterns. It includes journal and citation analysis to identify influential studies and emerging research hotspots. The ADR Analysis focuses on the safety profiles of GSK-3β inhibitors identified in the first part of the study, utilizing data from pharmacovigilance databases (FAERS, JADER, CVARD). This section systematically analyzes ADRs across various classification levels (SOC, PT, HLGT, HLT), conducts clinical significance assessments, and performs time-to-onset (TTO) and subgroup analysis to explore ADR variations by sex. The combination of both approaches provides a comprehensive understanding of the field’s development, safety profiles, and future directions.

### 2.2 Analysis of bibliometric analysis

#### 2.2.1 Data collection and retrieval strategy

The Core Collection of Web of Science (WoSCC) is a globally recognized, multidisciplinary bibliographic database comprising ten sub-collections and covering more than 12,000 prestigious academic journals. WoSCC provides comprehensive data essential for bibliometric analysis (23–25). Currently, CiteSpace processes data in WoSCC format, whereas data retrieved from other databases require format conversion before analysis. Consequently, all bibliometric data in this study were obtained directly from the WoSCC database (26).

To ensure consistency and avoid potential bias from subsequent literature updates, all records were retrieved on a fixed date, February 5, 2025. The applied search strategy was as follows: ALL = (“GSK-3β*” OR “GSK-3 beta*” OR “Glycogen synthase kinase-3 beta*” OR “GSK-3B*” OR “GSK3B*”) AND ALL = (“inhibitor*” OR “antagonist*”) AND TS = (“cancer” OR “tumor” OR “neoplasm” OR “carcinoma” OR “malignancy”) with a publication year constraint of 1999-2025. This search yielded a total of 2,006 potentially relevant publications.

To determine the final set of records for analysis, the following criteria were applied (1): exclusion of studies unrelated to the research topic (2); exclusion of non-English publications; and (3) exclusion of documents lacking a complete research process, such as retracted papers, editorial materials, early access publications, letters, and book chapters. Based on these criteria, relevant studies were retained while non-compliant records were excluded. Specifically, 55 publications—including retracted papers, editorials, early access articles, letters, and book chapters—were removed. After full-text screening, an additional 1,668 papers deemed irrelevant to the research topic were excluded. Ultimately, 283 publications were included in the final analysis. Each bibliographic record contained detailed information, including abstract, title, authors, keywords, country/region, institutions, journal, and references, etc.

#### 2.2.2 Statistical analysis

The data were exported in plain text format and imported into Biblioshiny for Bibliometrix (an online platform based on Bibliometrix 4.1.0). The processed Excel file, containing information such as titles, publication years, authors, countries/regions, affiliations, journals, keywords, keyword plus, citation counts, reference records, and abstracts, was generated for further analysis and interpretation. The statistical analysis in this study is based on the comprehensive table (**Supplementary Table 1**). The organized data were then imported into R-based Bibliometrix 4.1.0, VOSviewer (version 1.6.18), CiteSpace (version 5.8.R2), and Excel (2019 version) for statistical analysis and visualization.

Bibliometrix is an R-based bibliometric tool for statistical analysis and visualization, having been adopted in over a thousand bibliometric studies. Strategic maps were created with density index on the vertical axis and centrality index on the horizontal axis (27). A higher density index indicates greater maturity of a topic, while a higher centrality index suggests that the topic is closely connected with other themes and lies at the core of all research topics. Keyword co-occurrence density visualization was conducted using VOSviewer (27, 28). Each point on the map was color-coded according to the density of surrounding elements, with higher densities indicated by red and lower densities by blue. The density is positively correlated with the number and importance of surrounding elements. CiteSpace was employed for keyword clustering and timeline visualization (29, 30). A modularity Q > 0.3 and weighted average silhouette > 0.5 in CiteSpace indicate that the clustering results are robust. All radar charts, histograms, line graphs, and scatter plots were analyzed using Excel 2019. Violin plots were generated using GraphPad Prism 8. The journal impact factors were retrieved from the 2024 Journal Citation Reports (JCR). A p-value < 0.05 was considered statistically significant.

### 2.3 Analysis of adverse drug reactions database

#### 2.3.1 Data sources and processing

A retrospective analysis was performed a retrospective analysis of pharmacovigilance utilizing the FAERS database, JADER database and CVARD database. FAERS, a database that stores data regarding adverse events and medication errors reported to the agency. The dataset encompasses seven distinct data tables, patient demographic and administrative details (DEMO), information on drugs and biologics (DRUG), adverse events (REAC), patient outcomes (OUTC), sources of reports (RPSR), drug therapy start and end dates (THER), and indications for use or diagnoses (INDI). These tables collectively provide comprehensive information for analysis. This system is specifically designed to assist the FDA in conducting post-marketing safety surveillance for drugs and therapeutic biologics. With over 4 million voluntarily submitted reports, FAERS is recognized as the world’s largest collection of spontaneously reported adverse event data.

JADER, established in 2004, is composed of four main files: DEMO, DRUG, REAC, and HIST. These files collectively store detailed information on cases reported by pharmaceutical companies and medical institutions. The DEMO file features basic patient information, including sex, age, and weight. The DRUG file details the generic name of the medication, the route of administration, and the dates when the drug was administered. The REAC file documents the name of the adverse event, its outcome, and the date it occurred. Meanwhile, the HIST file provides information about the patient’s underlying medical conditions. Together, these files enable comprehensive monitoring and analysis of adverse events associated with medications.

CVARD, established in 1965, comprises reports submitted by healthcare professionals, consumers, manufacturers, and distributors. The database is managed by seven regional offices under the Canadian Vigilance Program, which oversees its operation. This program employs the database to conduct post-market safety monitoring of medications, ensuring their safety following market release.

After preprocessing, the FAERS dataset contained 232,066 AE reports in which a study drug was recorded as the primary suspect and 663,785 associated preferred terms (PTs) (**Figure 1**). The corresponding JADER dataset contained 22,115 AE reports and 20,436 PTs, and the CVARD dataset contained 123,973 AE reports and 103,188 PTs. These counts represent submitted reports and report-level terms; they do not measure the incidence of adverse events.

#### 2.3.2 Drug standardization and adverse event classification

Drug names were standardized using MedEx UIMA 1.3.8 to resolve naming inconsistencies and identify reports for the literature-derived study-drug panel. Reported adverse events (AEs) were classified with MedDRA version 27.1 by linking PTs to their corresponding system organ classes (SOCs). The analysis focused on reports in which a study drug was recorded as the primary suspected drug. Throughout the manuscript, the term AE is used for a spontaneously reported event; a disproportionality signal is not interpreted as a confirmed adverse drug reaction.

#### 2.3.3 Signal detection

The study-drug panel was derived from the literature search conducted for the bibliometric component and included all agents reported to exert direct or indirect effects on GSK-3β. The complete list of drugs included in the pharmacovigilance analysis is provided in **Supplementary Table 2**. To enable cross-database comparisons, drug names were standardized and harmonized across the included pharmacovigilance databases. The resulting panel was pharmacologically heterogeneous, comprising the direct ATP-competitive GSK-3 inhibitor 9-ING-41, lithium, which inhibits GSK-3 through both direct and indirect mechanisms, and several clinically used agents with reported GSK-3β-modulating activity. **Table 1** further summarizes the primary clinical indications of these agents and classifies the strength of evidence supporting their direct or indirect effects on GSK-3β. Agents supported by lower-certainty or predominantly indirect evidence were retained for exploratory signal detection but were not interpreted as selective GSK-3β inhibitors (31–34).

**Table 1:** Analyzed agents, primary clinical contexts, and strength of evidence for GSK-3β modulation.

Agent	Primary clinical indication / context	GSK-3β activity classification used for interpretation	Evidence and interpretive note
Elraglusib (9-ING-41)	Investigational anticancer agent	Direct ATP-competitive GSK-3β inhibitor	Direct target engagement is reported; nevertheless, target selectivity and downstream effects should be interpreted in the specific experimental context (31).
Lithium	Mood stabilizer for bipolar disorder	Direct and indirect GSK-3 inhibition	Competes with Mg2+ at GSK-3 and also regulates upstream pathways; it is not isoform-selective (32).
Valproate	Antiseizure drug and mood stabilizer	GSK-3β-modulating; direct inhibition reported in selected assays	GSK-3 inhibition has been reported in SH-SY5Y and biochemical assays, but the effect is not a basis for treating valproate as a selective GSK-3β inhibitor (33).
Olanzapine	Antipsychotic for schizophrenia and bipolar disorder	Direct GSK-3β inhibition reported in a recombinant-enzyme assay	An in-vitro recombinant GSK-3β IC50 of 91 nM was reported; its clinical pharmacology remains pleiotropic (34).
Caffeine	CNS stimulant	Lower-certainty GSK-3β-associated agent	No direct-inhibitor claim is made in this revision; retained from the original literature-derived screen and interpreted exploratorily.
Cimetidine	H2-receptor antagonist	Lower-certainty GSK-3β-associated agent	No direct-inhibitor claim is made in this revision; retained from the original literature-derived screen and interpreted exploratorily.
Ibuprofen	Non-steroidal anti-inflammatory analgesic	Lower-certainty GSK-3β-associated agent	No direct-inhibitor claim is made in this revision; retained from the original literature-derived screen and interpreted exploratorily.
Lidocaine	Local anesthetic / antiarrhythmic	Lower-certainty GSK-3β-associated agent	No direct-inhibitor claim is made in this revision; retained from the original literature-derived screen and interpreted exploratorily.

Disproportionality analysis was used to screen for drug-event pairs reported more frequently than expected. To improve robustness, two frequentist methods (reporting odds ratio [ROR] and proportional reporting ratio [PRR]) and two Bayesian methods (Bayesian confidence propagation neural network [BCPNN] and empirical Bayes geometric mean [EBGM]) were applied. **Supplementary Tables 3**, **S4** provide the formulas, 2×2 contingency tables, and positive-signal thresholds. A signal exceeding a threshold indicates disproportionate reporting, not a causal association or an incidence estimate. Analyses adjusted for available age, sex, weight, and report-source variables; residual confounding, differential reporting, and co-medication effects remain possible (22).

#### 2.3.4 Clinical significance analysis

Adverse events (AEs) with significant adverse reactions were prioritized based on clinical significance as follows:

First, important medical events (IMEs) and designated medical events (DMEs) were identified from the public list developed and updated by the EMA. IMEs are AEs with features of seriousness, while DMEs are rare, severe AEs with a high drug-attribution risk, which may pose safety concerns.

Second, IMEs and DMEs were prioritized using a semi-quantitative scoring system that evaluates five characteristics (**Supplementary Table 5**): reporting rate, signal stability, reported case fatality rate, clinical relevance. Scores of 0-2, 3-5, or 6–8 indicate weak, moderate, or strong clinical priority, respectively.

#### 2.3.5 TTO analysis

The TTO of AEs linked to GSK 3βGSK-3β inhibitors is calculated as the interval from the medication’s start date in the THER file (START_DT) to the AE’s onset date in the DEMO file (EVENT_DT). Cases with missing dates or AE onset dates predating the GSK 3βGSK-3β inhibitors’ start date were excluded. Accurate TTO data is crucial as the frequency of adverse events post-therapy initiation varies with the drug’s mechanism and can change over time, unlike non-drug-related events which occur at a steady rate. To evaluate these temporal patterns, the Weibull distribution test was used to assess changes in adverse event rates over time, indicating whether the risk increases or decreases. Our comprehensive TTO analysis included median, quartiles, extremes, and the Weibull distribution test (35, 36). The Weibull distribution is characterized by two parameters: scale (α) and shape (β). Here, only β is analyzed. If β <1 with a 95% confidence interval (CI) also <1, the risk decreases over time (early failure). If β≈1 with a CI including 1, the risk is constant (random failure). If β>1 with a CI excluding 1, the risk increases (wear-out failure). To ensure robust results, the Weibull test was applied to both the overall dataset and subgroups.

Compatible treatment-start and event-onset dates were unavailable in JADER and CVARD. Therefore, TTO analyses were restricted to FAERS and should not be interpreted as cross-database comparisons.

#### 2.3.6 Subgroup analysis

Sex-stratified analyses were performed at the SOC and PT levels. Because sex-specific data were unavailable in CVARD, no sex-stratified analysis was conducted for that database.

#### 2.3.7 statistical analysis

To evaluate differences in values across multiple groups, the Kruskal–Wallis test and Dunn’s test were employed. Data management was conducted using Microsoft Excel 2019. For statistical analysis and visualization, the R programming language (version 4.2.1) was utilized.

### 2.4 Gene expression omnibus database analysis

The transcriptomic data utilized in this study were sourced from the publicly available GEO database under accession number GSE290452, which profiles human induced pluripotent stem cell-derived neuronal cultures treated with lithium as a GSK-3β inhibitors versus untreated controls. The raw gene expression count matrix was processed and analyzed within the R statistical environment (v4.5.2). Low-expression genes were filtered out, retaining only those with counts ≥ 5 in at least two samples. Differential expression analysis between lithium-treated and control groups was performed using the DESeq2 package (v1.48.2) under a model design accounting for treatment condition. Significantly differentially expressed genes (DEGs) were defined using a threshold of p-value < 0.05 and an absolute log_2_ fold change > 1. To elucidate the biological functions and pathways associated with the identified DEGs, Gene Ontology (GO) enrichment analysis—covering biological processes, cellular components, and molecular functions—and Kyoto Encyclopedia of Genes and Genomes (KEGG) pathway analysis were conducted using the online bioinformatics platform Microbioinformatics (https://www.bioinformatics.com.cn), with terms considered significant at p < 0.05. Visualization of the results included a volcano plot generated with the ggplot2 package to display expression changes and statistical significance, and a heatmap created using the pheatmap package to depict normalized expression patterns of the top differentially expressed genes. All other data handling and graphical outputs were produced using standard functions within the R tidyverse ecosystem.

### 2.5 Experimental validation

#### 2.5.1 Cell culture, treatment, and key resources

Human neuroblastoma SH-SY5Y cells were cultured in Dulbecco’s Modified Eagle Medium/Nutrient Mixture F-12 (DMEM/F12; PM150312; Wuhan Procell Biotechnology Co., Ltd.) supplemented with 10% fetal bovine serum (FBS; XRFBS10-500; XR Biotechnolgies Co., Ltd., Hangzhou, China) and 1% penicillin–streptomycin solution (BC-CE-007;SenbeijiaBiological Technology Co., Ltd.). Cells were maintained at 37°C in a humidified incubator containing 5% CO_2_.For subculture, the culture medium was removed, and the cell monolayer was washed once with phosphate-buffered saline (PBS). Then, 2 mL of 0.25% trypsin–EDTA solution (SB-CR013N;Share-bio,Shang Hai, China) was added to each 10-cm culture dish [H802002;BaiDi Biotechnology Co., Ltd. (BDBIO)], and the cells were incubated at 37 °C for 2–3 min until they detached. Trypsinization was neutralized by adding an equal volume of complete culture medium containing 10% FBS. The cell suspension was transferred to a 15-mL centrifuge tube and centrifuged at 1,000 rpm for 5 min at room temperature. The supernatant was discarded, and the cell pellet was resuspended in fresh complete medium for further passage or in a serum-free cell cryopreservation medium (AC05L033;Shanghai Life-iLab Biotech Co., Ltd) for long-term storage. For cryopreservation, the cell density was adjusted to approximately 1–2 × 10^6^ cells/mL in the cryopreservation medium, and 1-mL aliquots were dispensed into sterile cryogenic vials (AC05-20, BIOLAND). The vials were placed in a controlled-rate freezing container and stored at –80 °C overnight, then transferred to liquid nitrogen for long-term maintenance.

For experimental validation, SH-SY5Y cells [SH-SY5Y cell lines were purchased from IMMOCELL (Xiamen, Fujian, China)] were treated with the GSK-3β inhibitor 9-ING-41 (T14066, Targetmol, USA). The 1 μM, 24-h condition was selected from the CCK-8 concentration-response experiment because viability remained approximately 90% of the untreated control, allowing molecular changes to be assessed without extensive cytotoxicity. A 24-h exposure has also been used in experimental studies of 9-ING-41 (31). Untreated cells served as controls.

#### 2.5.2 Cell viability assay

Cell viability was evaluated using a Cell Counting Kit-8 (CCK-8; CA1212; Solarbio Science & Technology Co., Ltd., Beijing, China) according to the manufacturer’s instructions. Briefly, SH-SY5Y cells were seeded into 96-well plates and treated with different concentrations of 9-ING-41 (0.25, 0.5, 1, 2, 5, and 10 μM) for 24 h. Subsequently, 10 μL of CCK-8 solution was added to each well and incubated for 2 h at 37°C. Absorbance was measured at 450 nm using a microplate reader. Cell viability was expressed as a percentage relative to the untreated control group.

#### 2.5.3 Quantitative real-time PCR

Total RNA was extracted using TRIzol Reagent (CW0580, CWBIO, China). RNA concentration and purity were determined using a NanoDrop 2000 spectrophotometer (Thermo Fisher Scientific).

Complementary DNA was synthesized using the Hifair^®^ III One Step RT-qPCR SYBR Green Kit for qPCR (11143ES70;Yeasen Biotechnology Co., Ltd., Shanghai, China). Quantitative real-time PCR was performed using Hieff^®^ qPCR SYBR Green Master Mix (11184ES;Yeasen Biotechnology Co., Ltd., Shanghai, China) on a QuantStudio™ 5 Real-Time PCR System (Applied Biosystems, Thermo Fisher Scientific).

The mRNA expression levels of GABRE, NPY, BRS3, KCNQ5, KCNH6, NLRP3, IL1B, CASP1, TNFA, IL6, CTNNB1, AXIN2, CCND1, and MYC were quantified. GAPDH was used as the internal reference gene. Relative gene expression levels were calculated using the 2^-ΔΔCt method.

#### 2.5.4 Intracellular ROS detection

Intracellular reactive oxygen species (ROS) levels were assessed using a Reactive Oxygen Species Assay Kit containing 2’-7’-dichlorodihydrofluorescein diacetate (DCFH-DA; ID31309; Solarbio Science & Technology Co., Ltd., Beijing, China). Following treatment with 1 μM 9-ING-41 for 24 h, SH-SY5Y cells were incubated with 10μM DCFH-DA at 37°C for 30 min in the dark. Cells were subsequently washed three times with serum-free medium to remove excess probe. Fluorescence intensity was measured using a fluorescence microplate reader (excitation: 488 nm, emission: 525 nm) and normalized to the control group.

#### 2.5.5 Statistical analysis of experimental data

Experimental data are presented as mean ± SEM. The CCK-8 concentration-response study used five biological replicates per condition. qRT-PCR assays used three independent biological experiments, whereas DCFH-DA assays included five measurements per group; technical replicates from the same biological experiment were averaged before analysis. A biological replicate denotes an independently seeded and treated culture, whereas a technical replicate denotes repeated measurements from the same biological sample. One-way ANOVA with Dunnett’s multiple-comparisons test was used for the concentration-response study; ordinary two-way ANOVA followed by Šídák’s multiple-comparisons test was used for each qRT-PCR panel; an unpaired two-tailed Student’s t-test was used for the DCFH-DA comparison. Analyses were performed with GraphPad Prism 10.0; two-sided P < 0.05 was considered statistically significant.

## 3 Results

### 3.1 Results of bibliometric analysis

#### 3.1.1 Annual publication trends and cumulative output of GSK-3β inhibitor studies

The number of studies on GSK-3β inhibitors has increased significantly over the past decade, reaching a peak around 2020. Although there has been a slight recent decline, the overall high level of publications suggests sustained interest in the field, and we anticipate that research activity may research activity may remain strong or rebound in the coming years. (**Supplementary Figure 1A**)To visually present the trend in publications, we performed a binomial fit a binomial fit was performed using Origin (Origin 2021; OriginLab Corporation), with the red dashed line representing the fitted trend. Although the search cutoff date was set for February 2025—meaning that not all relevant studies from 2025 were included—the overall trendline indicates a generally increasing pattern in publication volume over the past two decades (R² = 0.7105, suggesting a reasonably good model fit), as shown in **Supplementary Figure 1B**. Despite some recent fluctuations, the cumulative number of documents continues to rise steadily, reflecting sustained research interest in this field.

#### 3.1.2 Analysis of global output and international cooperation in the research of GSK-3β inhibitors

The country of relevant documents was based on the country of the corresponding author and the first author. A total of 61 countries contributed to research publications in this field, collectively producing 1171 documents. These included 19 countries from Asia (31%) and 26 from Europe (43%), highlighting the strong research presence across these two continents. The United States ranked first with 312 publications, followed by China (219) and Japan (106). The detailed country-wise distribution is shown in **Figure 2A**.

**Figure 2:**
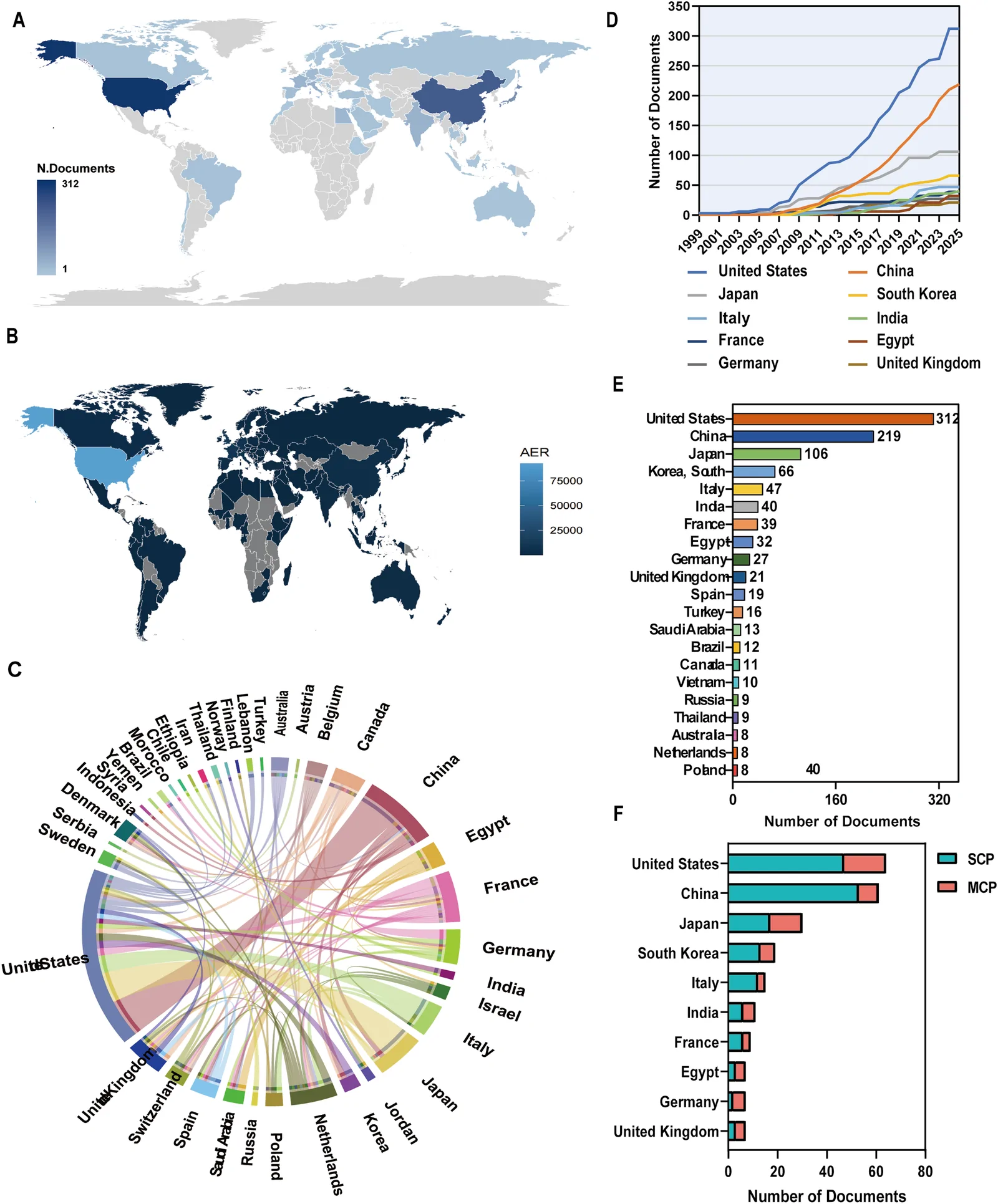
Global research output and adverse event reporting for GSK-3β inhibitors. **(A)** Geographic distribution of publications based on the country of the corresponding or first author, showing contributions from 61 countries, with the United States, China, and Japan as the top three producers. **(B)** Number of reported adverse events (AERs) in the FAERS database by country, with the United States reporting the highest number of cases (138,338, 59.6%), followed by the United Kingdom (17,011, 7.3%) and France (12,094, 5.2%). **(C)** Chord diagram illustrating international collaboration between countries, highlighting strong partnerships between China and the United States, and other notable collaborations involving the United States with Italy, Germany, Japan, and the United Kingdom. **(D)** Annual publication trends of top publishing countries over the past decade, with rapid growth observed in the United States and China. **(E)** Top 21 countries ranked by total publications. **(F)** Domestic (SCP) and international (MCP) collaboration indices of leading countries, showing that while international collaboration is common, most countries still produce more single-country publications. Overall, these data provide a comprehensive view of both the global research activity and safety reporting associated with GSK-3β inhibitors.

According to the international cooperation visualization map (**Figure 2C**), China and the United States exhibit the closest collaborative relationship, as indicated by the thickest connecting band. This is followed by strong partnerships between the United States and Italy, Germany, Japan, and the United Kingdom. In contrast, collaborations among other countries appear more dispersed and less frequent.

In the past decade, the United States and China have shown the most rapid growth in the number of publications in this field, with both countries demonstrating a marked upward trajectory since around 2010. Other countries such as Japan, South Korea, and Italy also exhibited steady increases in publication volume over the same period (**Figure 2D**).

Based on the number of publications, the United States leads the field with 312 documents, followed by China (219), Japan (106), and South Korea (66). Other notable contributors include Italy, India, France, and Egypt, each with more than 30 publications (**Figure 2E**).

As shown in **Figure 2F**, the United States and China lead in the total number of publications, with the United States demonstrating a notably higher proportion of multi-country publication (MCP) compared to China. Other countries such as Japan, South Korea, and India also showed varying degrees of international cooperation. Overall, most countries exhibited more single-country publications (SCP) than multi-country ones, indicating that domestic collaboration still dominates in this field.

International collaboration is common in this field, with an overall MCP rate of approximately 31.14%. The United States, as a leading research contributor, has collaborated extensively with both developed and developing countries. According to the data, 26.6% of U.S. publications involve international co-authorship. Similarly, China, Japan, South Korea, and several European countries have demonstrated varying degrees of global cooperation. Notably, countries such as France (71.4%), the United Kingdom (57.1%), and Egypt (57.1%) show particularly high proportions of internationally co-authored articles. Meanwhile, emerging economies including India, Egypt, and Iran are playing an increasingly active role. These collaborative efforts between developed and developing nations are essential for advancing global progress in this research area. The country of the document was based on the country of the corresponding author. If there were corresponding authors affiliated with institutions in different countries, the country of the first author shall prevail.

#### 3.1.3 Comparative analysis of GSK-3β inhibitors research between China and the United States

In order to further compare the differences between the publications and research of China and the United States, we conducted a more in-depth analysis of them.

Research on GSK-3β inhibitors have been primarily concentrated in China and the United States, while contributions from other countries were relatively limited and thus not further analyzed. As shown in the provincial-level heat map of China (**Figure 3A**), research efforts are mainly distributed across eastern and southern regions, with Shandong Province leading (9 publications), followed by Shanghai (8), Guangdong Province (6), Henan Province (5), and Sichuan Province (5), indicating regional clusters of scientific activity.

**Figure 3:**
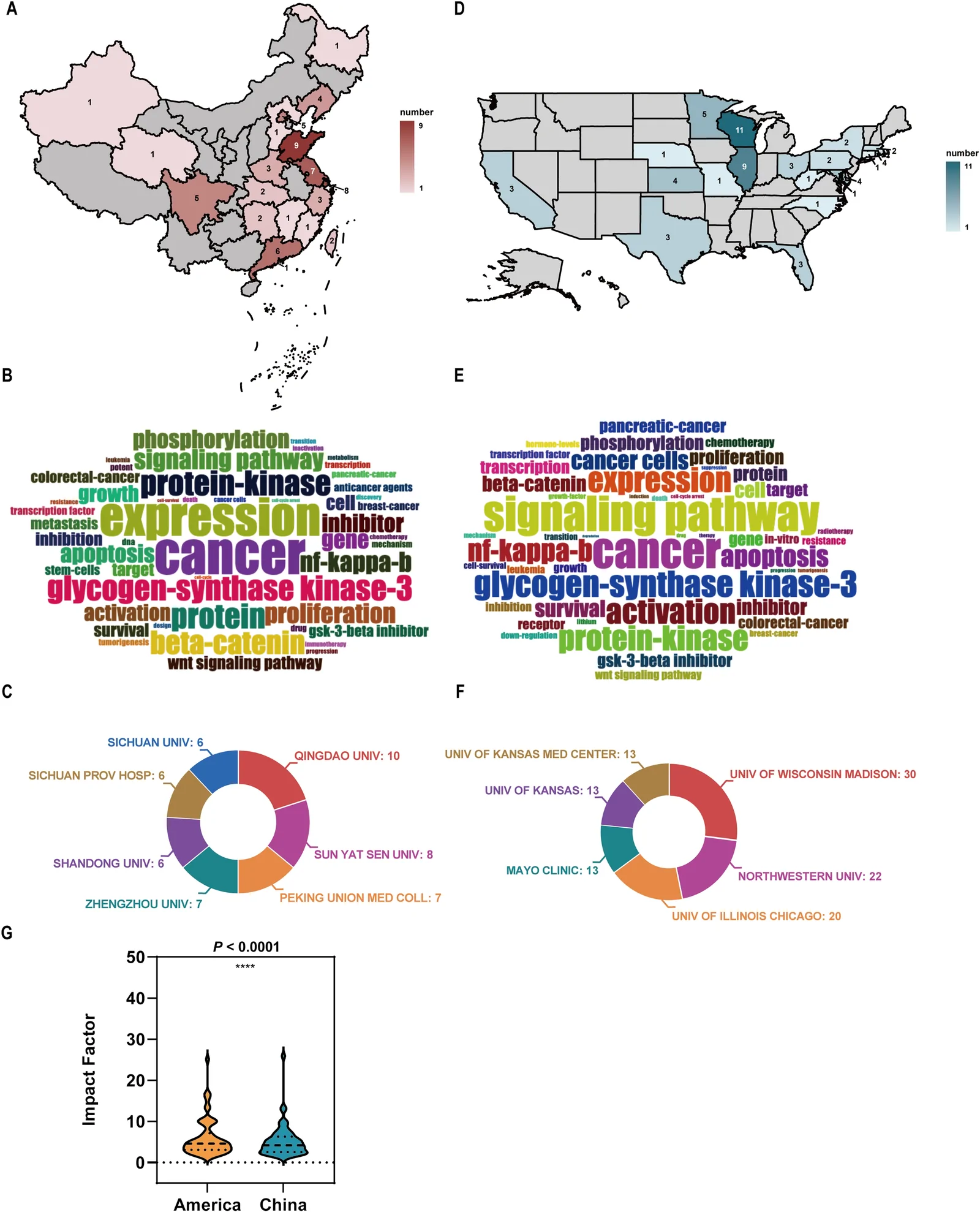
Comparative analysis of GSK-3β inhibitors research in China and the United States. **(A)** Provincial-level heat map of China showing the distribution of publications, with the highest research output concentrated in Shandong, Shanghai, Guangdong, Henan, and Sichuan provinces. **(B)** Word cloud of research topics in China, highlighting key terms such as stem cells, tumorigenesis, and pancreatic cancer. **(C)** Pie chart of the top five institutions in China for GSK-3β inhibitors research, with Qingdao University, Sun Yat Sen University, and Peking Union Medical College leading. **(D)** State-level heat map of the United States showing a dispersed distribution of publications, with Minnesota leading, followed by Illinois and several other states. **(E)** Word cloud of research topics in the United States, with greater emphasis on cancer cells, chemotherapy, *in-vitro* studies, and receptor-related mechanisms. **(F)** Pie chart of the top five institutions in the United States for GSK-3β inhibitors research, including University of Wisconsin Madison, Northwestern University, and University of Illinois Chicago. **(G)** Comparison of the average impact factor (IF) of articles from China and the United States, with U.S. publications showing a significantly higher average IF (5.93) compared to China (5.20), with a statistically significant difference (P < 0.0001). This figure provides a detailed comparison of regional publication distribution, institutional contributions, research focuses, and publication quality between the two countries.

The heat map of publications by U.S. states (**Figure 3D**) revealed a relatively dispersed distribution of research output. Minnesota contributed the highest number of publications (11), followed by Illinois (9), and several other states including California, Texas, and Florida (each with 3 publications). Notably, active contributions were observed not only along the East Coast, but also across the Midwest and Southern regions.

In the field of GSK-3β inhibitors research, both China and the United States share several major research hotspots, including cancer, apoptosis, signaling pathways, expression, glycogen synthase kinase-3, and protein kinases. However, some differences were observed between the two countries. China-specific hotspots emphasized terms such as stem-cells, tumorigenesis, and pancreatic cancer, whereas research in the United States showed greater focus on cancer cells, chemotherapy, *in-vitro* studies, and receptor-related mechanisms (**Figures 3B, E**).

The top institutions in China for GSK-3β inhibitors research included Qingdao University (10 publications), Sun Yat Sen University (8), Peking Union Medical College (7), Zhengzhou University (7), and Sichuan University, Sichuan Provincial Hospital, and Shandong University (each with 6 publications). In the United States, the leading institutions were University of Wisconsin Madison (30 publications), Northwestern University (22), University of Illinois Chicago (20), and University of Kansas, University of Kansas Medical Center, and Mayo Clinic (each with 13 publications) (**Figures 3C, F**).

Using IF as an indicator of publication quality, a significant difference was observed between articles from China and the United States. The average IF of American articles was 5.93, while the average IF of Chinese articles was 5.20, and the difference was statistically significant (P < 0.0001), as shown in **Figure 3G**.

#### 3.1.4 Key publications and citation analysis on GSK-3β inhibitors in cancer research

Research on the role of GSK-3β inhibitors in cancer biology has attracted extensive attention and citations. For example, Luo J and a team wrote a 2009 paper about how this enzyme helps cancer cells stay alive and multiply (37). They explained how bad enzyme activity stops cancer cells from dying. People cited this paper 369 times globally (**Figure 4B**) and 29 times in this specific group of studies (**Figure 4A**).

**Figure 4:**
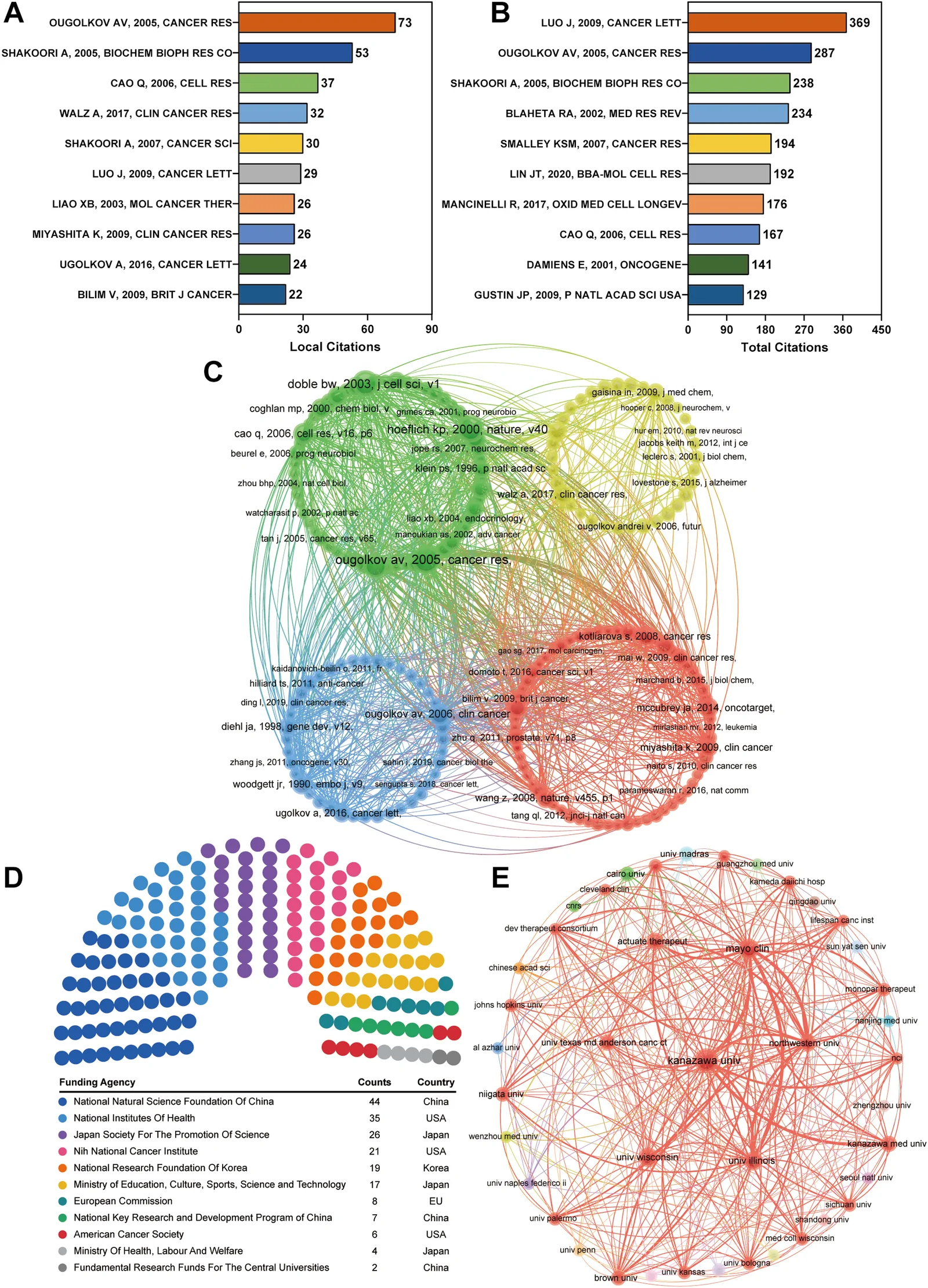
Key publications, citation analysis, funding support, and co-authorship network in GSK-3β inhibitors research. **(A)** Citation counts within the specific group of studies focusing on GSK-3β inhibitors in cancer research. This panel shows the number of local citations for key publications, offering insight into the impact of these studies within this particular research community. **(B)** Global citation counts for major publications on GSK-3β inhibitors. This chart highlights the total number of citations these influential papers have received worldwide, demonstrating the global recognition and importance of these contributions. **(C)** Citation relationships between the key literature on GSK-3β inhibitors in cancer research. This diagram shows the interconnections between pivotal works, reflecting how these studies reference each other and contribute to the scientific discourse in this field. **(D)** Top funding agencies supporting research on GSK-3β inhibitors. The National Natural Science Foundation of China is the leading funder, supporting 44 studies, followed by the National Institutes of Health (USA) with 35 studies, and the Japan Society for the Promotion of Science with 26 studies. Additional significant contributors include the NIH National Cancer Institute, the National Research Foundation of Korea, and the Ministry of Education, Culture, Sports, Science, and Technology of Japan. **(E)** Co-authorship network of 50 institutions involved in GSK-3β inhibitors research. The network is divided into multiple clusters, each represented by a different color. Kanazawa University is the most connected institution, serving as a key player in fostering collaborations across different research centers, underlining its central role in promoting inter-institutional cooperation and knowledge exchange within this area of research. ****p < 0.0001.

Some researchers want to know how to block the enzyme to treat sick patients. Ougolkov AV found that stopping GSK-3β kills tumor cells and halts their growth (38). He suggested it is a good target for new cancer drugs. His paper got 287 global hits and 73 local citations. Shakoori A also looked at how the enzyme joins with other cancer signals to make tumors grow (39). Other scientists cited that work 238 times globally and 53 times locally. Two years later, Shakoori tested this idea in animals with colon cancer. The results proved that giving drugs to block GSK-3β really does slow down tumor growth and causes the cancer cells to die (40).

Other scientists looked at specific types of cancer to see similar effects. Cao Q proved that GSK-3β controls how fast ovarian cancer cells multiply (41). That study has 167 global and 37 local citations. Smalley KSM studied melanoma cells. His team saw that blocking the enzyme turns on a specific path called p53, which forces the cancer cells to die (42). An older paper by Blaheta RA explained how a drug named valproic acid fights tumors. The drug changes the inner cell signals controlled by GSK-3β to stop the cancer from growing (43).

Some experts wrote review papers to collect all these facts. Walz A wrote about the different cancer paths the enzyme controls and why doctors should target it (44). Mancinelli R wrote a paper the same year about the ways this enzyme causes tumors and how it might help in the clinic (45).

All these popular papers share a clear message. The GSK-3β enzyme is a big reason why cancer cells survive and multiply. Making drugs to block this enzyme is a smart way to fight the disease. This past work gives scientists clear ideas for creating new treatments.

In addition, there were also rich citation relationships between these key literatures (**Figure 4C**).

#### 3.1.5 Key publications and citation analysis on GSK-3β inhibitors in cancer research

The top funding agencies supporting GSK-3β inhibitors research are shown in **Figure 4D**. The National Natural Science Foundation of China was the most active funder, supporting 44 studies, followed by the National Institutes of Health (USA, 35), and the Japan Society for the Promotion of Science (26). Other major contributors included the NIH National Cancer Institute, the National Research Foundation of Korea, and the Ministry of Education, Culture, Sports, Science and Technology of Japan.

Additionally, **Figure 4E** illustrates the network of co-authorship among institutions, which includes 50 institutions. The network is divided into several clusters, each represented by different colors. The Kanazawa University stands out as the most connected institution, forming extensive collaborative links with multiple research centers across different clusters, indicating its central role in promoting inter-institutional cooperation and knowledge exchange within this field.

#### 3.1.6 Author contributions and citation analysis in GSK-3β inhibitors research

H-index and citation metrics were calculated for authors represented in the 283 included publications. A total of 1,782 authors contributed to the analyzed records, with a mean of 6.30 authors per article.

Among these authors, the top three in terms of publication count were Minamoto Toshinari (16 papers), Giles Francis J (11), and Kunnimalaiyaan Muthusamy and Ugolkov Andrey (both 9 papers) (**Supplementary Figure 2A**). The top three authors with the highest local h-index were Minamoto Toshinari (h-index = 10), Giles Francis J (9)., and Kunnimalaiyaan Muthusamy and Ugolkov Andrey (both 8) (**Supplementary Figure 2B**). The most locally cited authors were Minamoto Toshinari (143 citations), Billadeau D.D (126), and Ugolkov Andrey (116) (**Supplementary Figure 2C**).

The heat map of the annual publication volume of the top 20 authors is shown in **Supplementary Figure 2D**.

The author co-citation network is shown in **Supplementary Figure 2E**. The central authors in the field of GSK-3β inhibitors research include Andrei V. Ougolkov (Mayo Clinic College of Medicine, USA), Richard S. Jope (University of Miami, USA), Abbas Shakoori (Kanazawa University, Japan), Eléonore Beurel (University of Miami, USA), Bradley W. Doble (McMaster University, Canada), and Hoeflich KP (Ontario Cancer Institute/Princess Margaret Hospital, Canada). These authors appear as key nodes in the network, demonstrating their substantial influence in the relevant research areas.

A total of 1873 researchers contributed to the publication of 2127 studies. Among them, 1729 (92.31%) researchers published only one study, contributing 81.29% (1729/2127) of the total publication output. A further 105 (5.61%) researchers published 2 studies, accounting for 9.87% (210/2127) of the total output. According to the law developed by Price (46), the minimum threshold for core authors is 3 published papers. A total of 39 (2.09%) researchers reached this threshold, contributing 188 (8.84%) articles. However, this still does not meet the core author output threshold (>50%) as stipulated by the law developed by Price, as shown in **Supplementary Figure 2F**.

#### 3.1.7 Collaboration patterns, journal impact, and citation trends in GSK-3β inhibitor research

The journal co-citation network showed that papers in the field of GSK-3β inhibitors were primarily published in two types of journals (**Supplementary Figure 3A**). The red nodes represent journals focusing on biological and chemical sciences, while the blue nodes are predominantly medical and clinical journals. The abundant co-citations between these two clusters highlight the interdisciplinary nature of GSK-3β inhibitors research, bridging chemical and biological studies with clinical applications in cancer therapy.

**Supplementary Figure 3B** shows that Cancer Research published the highest number of articles on the subject (10 publications, 2024 IF = 16.6), followed by PLoS One (10 publications, 2024 IF = 2.6), and Clinical Cancer Research (7 publications, 2024 IF = 10.2). The journals with the fourth largest number of publications, each publishing 5 articles, included Scientific Reports (2024 IF = 3.9), Biochem Bioph Res Co (2024 IF = 2.2), and BMC Cancer (2024 IF = 3.4). It is noteworthy that all the top journals by publication volume belong to the Q1 division (2024 JIF quartile), and their impact factors are all above 2. These data indicate that the most productive journals in this dataset were predominantly Q1 journals with impact factors above 2.

On the other hand, the top five journals cited articles under study are: Cancer Research (603 articles, 2024 IF = 16.6), J Biol Chem (503 articles, 2024 IF = 3.9), Clin Cancer Res (323 articles, 2024 IF = 10.2), Oncogene (312 articles, 2024 IF = 7.3), and Nature (307 articles, 2024 IF = 48.5) (**Supplementary Figure 3C**). The top 20 journals include many prestigious journals in the field, such as Nature, Science, Cell, Advanced Materials, and Nature Reviews Cancer, reflecting that the theoretical foundation of GSK-3β inhibitors is robust and well-recognized by high-impact journals.

In the past few years, Cancer Research, Plos one and Clinical Cancer Research have experienced the fastest growth in the number of articles published in related fields, with a significant increase starting around 2020 (**Supplementary Figure 3D**).

**Supplementary Figure 3E** illustrates the core journals according to Bradford’s law, which are represented in the shaded area. The core sources, including “Cancer Research” and “PLOS One,” are the journals with the highest number of articles, contributing significantly to the publication output in this field. These core sources make up the majority of the publications, aligning with Bradford’s law, where a small number of journals account for a large proportion of the total research output.

#### 3.1.8 Keyword evolution and research trends in GSK-3β inhibitors

The cloud map of keywords (**Figure 5A**) shows that key research directions for the application of nanomaterials in tumor immunotherapy include cancer, protein kinase, glycosylation synthesis kinase-3, apoptosis, and expression. The keyword density map (**Supplementary Figures 4B–E**) further shows that in addition to the aforementioned topics, immune checkpoint blockade, chemotherapy, metastasis, biological evaluation, and other crucial areas of study were also prevalent research themes across the years (2019–2022).

**Figure 5:**
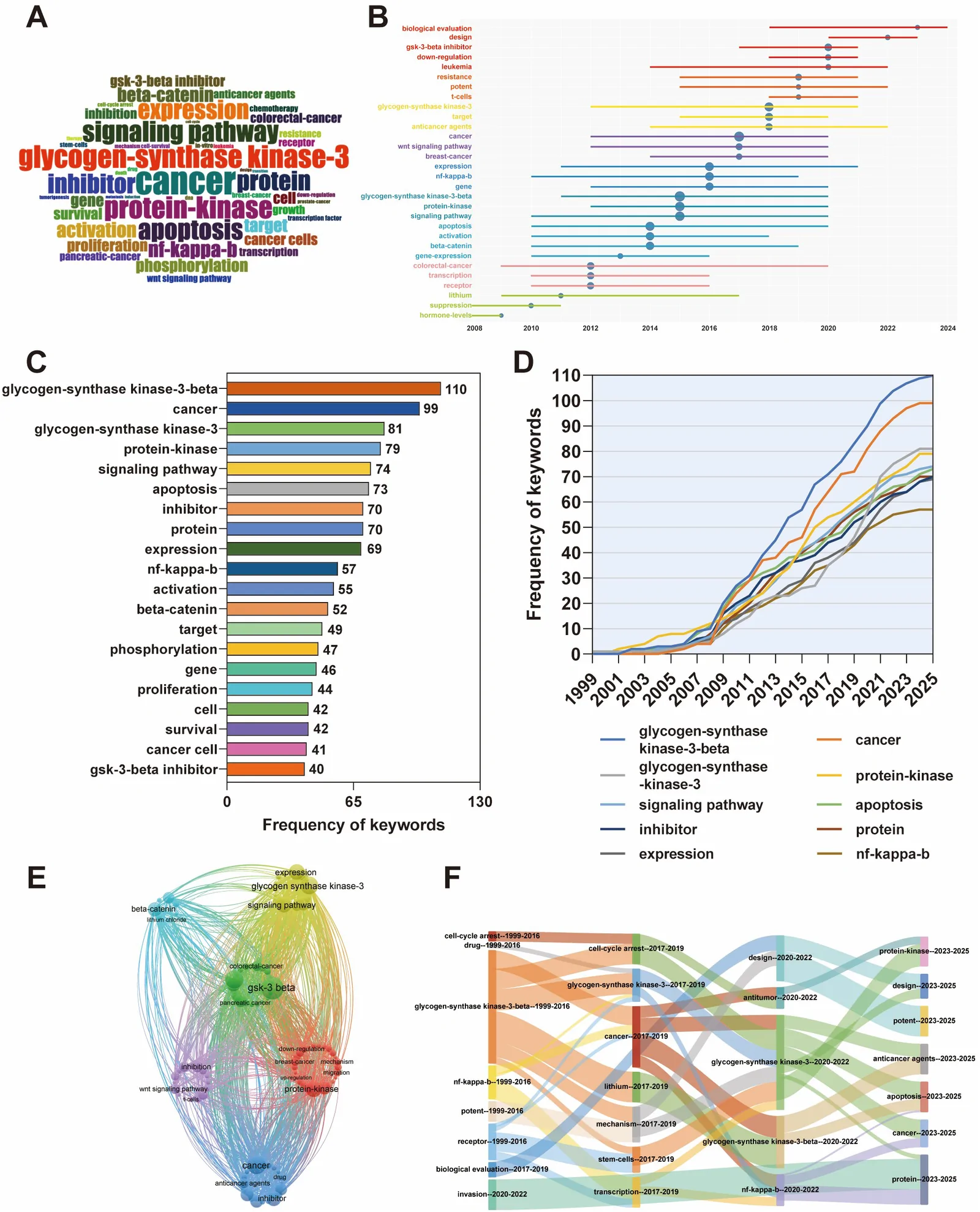
Comprehensive analysis of keyword trends in GSK-3β inhibitors research. **(A)** Word cloud of key research topics in the application of GSK-3β inhibitors. The cloud highlights cancer, protein kinase, glycogen-synthase kinase-3, apoptosis, and expression as dominant themes. **(B)** Annual keyword evolution chart showing the shift in focus across the years. In 2022, keywords like glycogen-synthase kinase-3, protein kinase, and apoptosis dominated; in 2021, terms like colorectal cancer, receptor, lithium, and suppression emerged; in 2020, cancer, expression, and glycogen-synthase kinase-3-beta were prevalent; and in 2019, topics like gene expression, beta-catenin, cancer, and activation were emphasized. **(C)** Top 20 keywords with the highest frequency of occurrence in the literature, with glycogen-synthase kinase-3-beta, cancer, and glycogen-synthase kinase-3 being the most frequently mentioned. **(D)** Annual term frequency line chart showing the rapid growth in the usage of keywords such as glycogen-synthase kinase-3-beta, cancer, protein kinase, and signaling pathways in recent years. **(E)** Keyword co-occurrence network visualized using VOSviewer, highlighting key clusters of research topics. Central nodes include GSK-3β, expression, glycogen synthase kinase-3, signaling pathway, and cancer, with distinct clusters emerging around these themes. **(F)** Sankey diagram illustrating the changes in keyword usage over time and their internal connections. Persistent topics like glycogen-synthase kinase-3-beta, cancer, and nf-kappa-b remain central (2017–2019), while emerging keywords such as anticancer agents and apoptosis gain attention (2023–2025).

The annual main keywords evolution chart reveals (**Figure 5B**) that in 2022, the main keywords included glycogen-synthase kinase-3, protein kinase, and apoptosis; in 2021, keywords such as colorectal cancer, receptor, lithium, and suppression emerged; in 2020, the keywords cancer, expression, and glycogen-synthase kinase-3-beta were prominent. In 2019, key topics included gene expression, beta-catenin, cancer, and activation. Earlier keywords were focused on target, nf-kappa-b, and signaling pathway.

The top five keywords with the highest frequency of occurrence are (**Figure 5C**): glycogen-synthase kinase-3-beta (110), cancer (99), glycogen-synthase kinase-3 (81), protein-kinase (79), and signaling pathway (74).

The annual term frequency line chart of keywords included in the literature showed that the usage of keywords like glycogen-synthase kinase-3-beta, cancer, protein-kinase, and signaling pathway has grown rapidly in recent years (**Figure 5D**).

The co-occurrence networks of keywords are visualized using VOSviewer (**Figure 5E**). The terms with high frequency are represented as larger nodes, and nodes of the same color signify closer associations. In this visualization, the central nodes include the following key terms: GSK-3β, expression, glycogen synthase kinase-3, signaling pathway, and cancer. VOSviewer automatically grouped keywords with similar themes into five distinct clusters. Cluster 1, depicted in green, is associated with the concepts of GSK-3β and signaling pathways. Cluster 2, shown in red, is linked to protein kinases and cancer-related terms. Cluster 3, represented in yellow, covers concepts such as colorectal cancer and beta-catenin. Cluster 4, illustrated in blue, is related to cancer cells, inhibition, and Wnt signaling pathways. Finally, Cluster 5, shown in purple, pertains to the terms drug inhibitors and targeting therapies.

As shown in **Figure 5F**, some studies have been enduring in recent years, such as glycogen-synthase kinase-3-beta (2017–2019), cancer (2017–2019), and nf-kappa-b (2017–2019), all of which have consistently remained central topics in the field. In addition, newer topics like anticancer agents (2023–2025) and apoptosis (2023–2025) are gaining increasing attention, as seen in the trend of emerging keywords in recent years.

The strategic diagram of the sub-period (**Supplementary Figure 4A**) shows that keywords such as glycogen-synthase kinase-3-beta, cancer, glycogen-synthase kinase-3, apoptosis, and protein-kinase are located in the Motor Themes quadrant, indicating that these topics are the core themes with high relevance and maturity. In addition, GSK-3β inhibitor, anticancer agents, therapy, and radiotherapy are also positioned in this quadrant, highlighting their ongoing importance. On the other hand, drug, metastasis, and cell-cycle arrest are located in the Emerging or Declining Themes quadrant, suggesting that while these topics have been explored, they may currently be experiencing a period of lower intensity in research (47, 48).

#### 3.1.9 Impact factor trends and collaboration patterns in GSK-3β inhibitor research

Correlation analysis showed that the impact factor of a study was positively correlated with the number of citations (**Supplementary Figure 5A**) and the number of references (**Supplementary Figure 5B**).

Based on the data shown in **Supplementary Figure 5C**, the average number of authors for papers with an impact factor greater than 10 (IF > 10) was significantly higher than that of papers with an impact factor of 10 or less (IF ≤ 10). The violin plot illustrates that papers with higher impact factors tend to have a broader distribution of authors, with many studies having a larger number of authors, whereas papers with lower impact factors show a more concentrated distribution with fewer authors on average. This difference was found to be statistically significant (P < 0.0001), suggesting that studies published in higher-impact journals often involve more collaborative efforts.

The average impact factor of articles published in recent years has significantly increased compared to those published before 2015 (**Supplementary Figure 5D**). This rise in impact factor is likely related to the growing trend of larger author teams and more collaborative research, particularly evident after 2018, as shown in the increasing number of authors per paper and the corresponding impact factor growth.

### 3.2 Results of adverse drug reactions database

#### 3.2.1 Descriptive characteristics

This study included data spanning from 2004 to 2024 from the FAERS and JADER databases, and data ranging from 1990 to 2023 from the CVARD database.

The demographic characteristics of AEs associated with GSK-3β inhibitors are presented in **Table 2**. In the FAERS database, the number of reports with determined gender was 190,825, including 87,165 male reports (37.6%) and 103,660 female reports (44.7%). The number of reports with age information was 127,876, with 16,151 (7.0%) under 18 years old, 72,197 (31.1%) between 18 and 64 years old, 35,746 (15.4%) between 65 and 85 years old, and 3,782 (1.6%) over 85 years old. Weight data were available for 46,221 reports, with the 50–100 kg group comprising the largest number at 33,772 (14.6%), followed by those under 50 kg at 7,558 (3.3%) and 100 kg or above at 4,891 (2.1%).

**Table 2:** Demographic characteristics of ADEs reported in the FAERS, JADER and CVARD database with GSK 3β inhibitors as the primary suspect drug.

Database	FAERS	JADER	CVARD
Case number	232066	12297	22569
Sex			
Female	103660 (44.7%)	6042 (49.1%)	11431 (50.6%)
Male	87165 (37.6%)	5637 (45.8%)	10172 (45.1%)
Unknown	41241 (17.8%)	618 (5.0%)	966 (4.3%)
Age			
<18	16151 (7.0%)	1758 (14.30%)	1663 (7.4%)
>85	3782 (1.6%)	122 (0.99%)	385 (1.7%)
18∼64	72197 (31.1%)	7743 (62.97%)	10753 (47.6%)
65∼85	35746 (15.4%)	1999 (16.26%)	5334 (23.6%)
Unknown	104190 (44.9%)	675 (5.49%)	4434 (19.6%)
Weight			
<50 kg	7558 (3.3%)	1856 (15.09%)	687 (3.0%)
≥100 kg	4891 (2.1%)	59 (0.48%)	584 (2.6%)
50∼100 kg	33772 (14.6%)	2596 (21.11%)	4150 (18.4%)
Unknown	185845 (80.1%)	7786 (63.32%)	17148 (76.0%)

In the JADER database, the number of reports with determined gender was 11,679, including 6,042 female reports (49.1%) and 5,637 male reports (45.8%). The number of reports with age information was 11,622, with 1,758 (14.30%) under 18 years old, 7,743 (62.97%) between 18 and 64 years old, 1,999 (16.26%) between 65 and 85 years old, and 122 (0.99%) over 85 years old. Weight data were available for 4,511 reports, with the 50–100 kg group comprising the largest number at 2,596 (15.09%), followed by those under 50 kg at 1,856 (15.09%) and 100 kg or above at 59 (0.48%).

In the CVARD database, the number of reports with determined gender was 21,603, including 11,431 female reports (50.6%) and 10,172 male reports (45.1%). The number of reports with age information was 18,135, with 1,663 (7.4%) under 18 years old, 10,753 (47.6%) between 18 and 64 years old, 5,334 (23.6%) between 65 and 85 years old, and 385 (7.4%) over 85 years old. Weight data were available for 5,421 reports, with the 50–100 kg group comprising the largest number at 4,150 (18.4%), followed by those under 50 kg at 687 (3.0%) and 100 kg or above at 584 (2.6%).

Through a combined analysis of the three databases, the analysis identified that the highest incidence of adverse drug events occurred in female, in individuals aged 18–64 years and with a weight of 50–100 kg.

**Supplementary Figures 1C–H** illustrates the annual reporting of adverse reaction event reports associated with GSK-3β inhibitors. Reports on adverse reactions of GSK-3β inhibitors drugs have increased particularly rapidly over the past decade, reaching their peak around 2020, which is consistent with the results of bibliometric studies.

In the FAERS database, the country with the highest number of reported Adverse Event Reports (AERs) was the United States, accounting for 138,338cases (59.6%), followed by United Kingdom, 17,011 cases (7.3%), France, 12,094 cases (5.2%) (**Figure 2B**).

#### 3.2.2 Signal detection at the SOC level

Across FAERS, JADER, and CVARD, 27 SOCs showed at least one disproportionality signal for the literature-derived study-drug panel (**Supplementary Figure 6**). Nervous-system disorders were among the higher-reporting and higher-ROR SOCs in all three databases. The most frequently reported neurological PTs differed by database: dizziness and somnolence were prominent in FAERS and CVARD, whereas neuroleptic malignant syndrome, altered state of consciousness, and seizure were prominent in JADER. Blood and lymphatic system reports also recurred across databases, with anemia, neutropenia, and thrombocytopenia prominent in FAERS and CVARD and thrombocytopenia, pancytopenia, and agranulocytosis prominent in JADER. These patterns identify cross-database consistencies and database-specific signals, but do not demonstrate causal, class-wide toxicity. Detailed SOC-level results are shown in **Tables 3**–**5**; **Supplementary Figure 6**.

**Table 3:** The signal strength of AERs for GSK 3β inhibitors at the SOC level in the FAERS database.

SOC	Case number	ROR (95%Cl)	PRR (chi-square value)	EBGM (EBGM05)	IC (IC025)
General disorders and administration site conditions	111975	0.95 ( 0.95 - 0.96 )	0.96 ( 217.09 )	0.96 ( 0.96 )	-0.06 ( -0.07 )
Nervous system disorders	76031	1.39 ( 1.38 - 1.41 )	1.35 ( 7393.9 )	1.34 ( 1.34 )	0.43 ( 0.41 )
Injury, poisoning and procedural complications	73093	1.18 ( 1.17 - 1.19 )	1.16 ( 1755.51 )	1.16 ( 1.15 )	0.21 ( 0.2 )
Gastrointestinal disorders	70121	1.26 ( 1.25 - 1.27 )	1.23 ( 3258.08 )	1.23 ( 1.22 )	0.29 ( 0.28 )
Psychiatric disorders	54926	1.5 ( 1.48 - 1.51 )	1.45 ( 8128.89 )	1.45 ( 1.44 )	0.53 ( 0.52 )
Investigations	38912	0.94 ( 0.93 - 0.95 )	0.94 ( 161.74 )	0.94 ( 0.93 )	-0.09 ( -0.1 )
Respiratory, thoracic and mediastinal disorders	32200	1.01 ( 1 - 1.02 )	1.01 ( 2.15 )	1.01 ( 1 )	0.01 ( -0.01 )
Skin and subcutaneous tissue disorders	30991	0.85 ( 0.84 - 0.86 )	0.86 ( 763.33 )	0.86 ( 0.85 )	-0.22 ( -0.24 )
Metabolism and nutrition disorders	18491	1.3 ( 1.28 - 1.32 )	1.29 ( 1227.53 )	1.29 ( 1.27 )	0.36 ( 0.34 )
Infections and infestations	18325	0.5 ( 0.49 - 0.51 )	0.52 ( 8767.8 )	0.52 ( 0.51 )	-0.95 ( -0.97 )
Cardiac disorders	17920	1.02 ( 1 - 1.03 )	1.01 ( 3.97 )	1.01 ( 1 )	0.02 ( 0 )
Musculoskeletal and connective tissue disorders	16721	0.46 ( 0.45 - 0.46 )	0.47 ( 10455.08 )	0.47 ( 0.47 )	-1.08 ( -1.1 )
Renal and urinary disorders	14804	1.21 ( 1.19 - 1.23 )	1.21 ( 528.74 )	1.2 ( 1.19 )	0.27 ( 0.24 )
Product issues	12772	1.21 ( 1.19 - 1.24 )	1.21 ( 466.62 )	1.21 ( 1.19 )	0.27 ( 0.25 )
Vascular disorders	11736	0.81 ( 0.79 - 0.82 )	0.81 ( 516.28 )	0.81 ( 0.8 )	-0.3 ( -0.32 )
Immune system disorders	11200	1.52 ( 1.49 - 1.55 )	1.51 ( 1929.75 )	1.5 ( 1.48 )	0.59 ( 0.56 )
Eye disorders	10237	0.76 ( 0.74 - 0.77 )	0.76 ( 785.89 )	0.76 ( 0.75 )	-0.39 ( -0.42 )
Blood and lymphatic system disorders	9362	0.82 ( 0.8 - 0.83 )	0.82 ( 381.7 )	0.82 ( 0.81 )	-0.29 ( -0.32 )
Hepatobiliary disorders	7451	1.22 ( 1.2 - 1.25 )	1.22 ( 295.95 )	1.22 ( 1.19 )	0.28 ( 0.25 )
Congenital, familial and genetic disorders	7409	3.79 ( 3.7 - 3.88 )	3.76 ( 14378.02 )	3.64 ( 3.57 )	1.86 ( 1.83 )
Surgical and medical procedures	3535	0.38 ( 0.37 - 0.4 )	0.39 ( 3489.26 )	0.39 ( 0.38 )	-1.36 ( -1.41 )
Pregnancy, puerperium and perinatal conditions	2921	1.02 ( 0.98 - 1.06 )	1.02 ( 1.2 )	1.02 ( 0.99 )	0.03 ( -0.02 )
Reproductive system and breast disorders	2904	0.53 ( 0.51 - 0.54 )	0.53 ( 1234.1 )	0.53 ( 0.51 )	-0.92 ( -0.97 )
Ear and labyrinth disorders	2778	0.96 ( 0.93 - 1 )	0.96 ( 3.58 )	0.97 ( 0.94 )	-0.05 ( -0.11 )
Neoplasms benign, malignant and unspecified (incl cysts and polyps)	2753	0.15 ( 0.15 - 0.16 )	0.15 ( 13074.94 )	0.16 ( 0.15 )	-2.68 ( -2.74 )
Social circumstances	2140	0.73 ( 0.7 - 0.76 )	0.73 ( 207.35 )	0.74 ( 0.71 )	-0.44 ( -0.51 )
Endocrine disorders	2077	1.22 ( 1.17 - 1.28 )	1.22 ( 81.52 )	1.22 ( 1.17 )	0.28 ( 0.22 )

**Table 4:** The signal strength of AERs for GSK 3β inhibitors at the SOC level in the JADER database.

SOC	Case number	ROR (95%Cl)	PRR (chi-square value)	EBGM (EBGM05)	IC (IC025)
Nervous system disorders	3609	2.09 ( 2.02 - 2.17 )	1.9 ( 1658.11 )	1.88 ( 1.81 )	0.91 ( -0.76 )
Injury, poisoning and procedural complications	1762	3.24 ( 3.09 - 3.41 )	3.05 ( 2403.46 )	2.97 ( 2.83 )	1.57 ( -0.1 )
Skin and subcutaneous tissue disorders	1716	1.41 ( 1.35 - 1.49 )	1.38 ( 187.4 )	1.37 ( 1.31 )	0.46 ( -1.21 )
Investigations	1670	0.84 ( 0.8 - 0.89 )	0.85 ( 45.16 )	0.86 ( 0.81 )	-0.22 ( -1.89 )
Metabolism and nutrition disorders	1302	1.51 ( 1.43 - 1.6 )	1.48 ( 207.89 )	1.47 ( 1.39 )	0.56 ( -1.11 )
Psychiatric disorders	1247	3.19 ( 3 - 3.38 )	3.05 ( 1688.25 )	2.97 ( 2.8 )	1.57 ( -0.09 )
Gastrointestinal disorders	1078	0.65 ( 0.61 - 0.69 )	0.67 ( 190.08 )	0.67 ( 0.63 )	-0.57 ( -2.24 )
Renal and urinary disorders	1005	1.27 ( 1.2 - 1.36 )	1.26 ( 55.46 )	1.26 ( 1.18 )	0.33 ( -1.34 )
General disorders and administration site conditions	955	0.7 ( 0.65 - 0.74 )	0.71 ( 119.67 )	0.71 ( 0.67 )	-0.49 ( -2.15 )
Cardiac disorders	856	1.02 ( 0.95 - 1.09 )	1.02 ( 0.22 )	1.02 ( 0.95 )	0.02 ( -1.64 )
Hepatobiliary disorders	836	1.01 ( 0.94 - 1.08 )	1.01 ( 0.04 )	1.01 ( 0.94 )	0.01 ( -1.66 )
Respiratory, thoracic and mediastinal disorders	739	0.48 ( 0.44 - 0.51 )	0.49 ( 409.37 )	0.5 ( 0.46 )	-1.01 ( -2.67 )
Blood and lymphatic system disorders	624	0.45 ( 0.41 - 0.48 )	0.46 ( 414.31 )	0.47 ( 0.43 )	-1.1 ( -2.77 )
Immune system disorders	594	0.95 ( 0.87 - 1.03 )	0.95 ( 1.52 )	0.95 ( 0.88 )	-0.07 ( -1.74 )
Infections and infestations	570	0.32 ( 0.3 - 0.35 )	0.34 ( 787.72 )	0.34 ( 0.32 )	-1.54 ( -3.21 )
Musculoskeletal and connective tissue disorders	501	0.91 ( 0.83 - 0.99 )	0.91 ( 4.41 )	0.91 ( 0.83 )	-0.13 ( -1.8 )
Congenital, familial and genetic disorders	335	7.09 ( 6.34 - 7.94 )	6.99 ( 1578.18 )	6.48 ( 5.79 )	2.7 ( 1.03 )
Vascular disorders	330	0.61 ( 0.54 - 0.68 )	0.61 ( 82.31 )	0.62 ( 0.55 )	-0.7 ( -2.37 )
Endocrine disorders	275	0.81 ( 0.72 - 0.92 )	0.82 ( 11.55 )	0.82 ( 0.73 )	-0.29 ( -1.96 )
Eye disorders	157	0.5 ( 0.43 - 0.59 )	0.51 ( 76.15 )	0.51 ( 0.44 )	-0.97 ( -2.64 )
Neoplasms benign, malignant and unspecified (incl cysts and polyps)	88	0.1 ( 0.08 - 0.13 )	0.11 ( 669.06 )	0.11 ( 0.09 )	-3.19 ( -4.85 )
Pregnancy, puerperium and perinatal conditions	70	0.88 ( 0.69 - 1.11 )	0.88 ( 1.17 )	0.88 ( 0.69 )	-0.18 ( -1.85 )
Reproductive system and breast disorders	49	0.62 ( 0.47 - 0.82 )	0.62 ( 11.59 )	0.62 ( 0.47 )	-0.69 ( -2.36 )
Surgical and medical procedures	28	0.33 ( 0.23 - 0.48 )	0.33 ( 38.03 )	0.33 ( 0.23 )	-1.59 ( -3.25 )
Ear and labyrinth disorders	21	0.46 ( 0.3 - 0.7 )	0.46 ( 13.56 )	0.46 ( 0.3 )	-1.12 ( -2.79 )
Social circumstances	16	1.53 ( 0.93 - 2.51 )	1.53 ( 2.86 )	1.52 ( 0.92 )	0.6 ( -1.07 )
Product issues	3	0.26 ( 0.08 - 0.81 )	0.26 ( 6.31 )	0.26 ( 0.08 )	-1.93 ( -3.6 )

**Table 5:** The signal strength of AERs for GSK 3β inhibitors at the SOC level in the CVARD database.

SOC	Case number	ROR (95%Cl)	PRR (chi-square value)	EBGM (EBGM05)	IC (IC025)
General disorders and administration site conditions	17679	0.78 ( 0.77 - 0.8 )	0.82 ( 850.48 )	0.83 ( 0.81 )	-0.28 ( -1.94 )
Gastrointestinal disorders	11188	1.09 ( 1.07 - 1.12 )	1.08 ( 78.59 )	1.08 ( 1.06 )	0.11 ( -1.55 )
Nervous system disorders	10885	1.48 ( 1.45 - 1.51 )	1.43 ( 1463.38 )	1.41 ( 1.38 )	0.5 ( -1.17 )
Investigations	10265	1.31 ( 1.28 - 1.33 )	1.28 ( 641.33 )	1.27 ( 1.24 )	0.34 ( -1.33 )
Respiratory, thoracic and mediastinal disorders	7774	1.34 ( 1.31 - 1.37 )	1.31 ( 587.17 )	1.3 ( 1.27 )	0.38 ( -1.29 )
Psychiatric disorders	7281	1.69 ( 1.65 - 1.73 )	1.64 ( 1820.41 )	1.61 ( 1.57 )	0.69 ( -0.98 )
Skin and subcutaneous tissue disorders	6327	1.04 ( 1.01 - 1.06 )	1.03 ( 7.52 )	1.03 ( 1.01 )	0.05 ( -1.62 )
Injury, poisoning and procedural complications	5451	0.75 ( 0.73 - 0.77 )	0.76 ( 424.28 )	0.77 ( 0.75 )	-0.38 ( -2.05 )
Musculoskeletal and connective tissue disorders	4312	0.59 ( 0.57 - 0.61 )	0.61 ( 1155.98 )	0.61 ( 0.6 )	-0.7 ( -2.37 )
Metabolism and nutrition disorders	4031	2.6 ( 2.52 - 2.69 )	2.54 ( 3536.58 )	2.42 ( 2.35 )	1.28 ( -0.39 )
Infections and infestations	3546	0.49 ( 0.47 - 0.51 )	0.51 ( 1782.84 )	0.52 ( 0.5 )	-0.96 ( -2.62 )
Cardiac disorders	2393	1.21 ( 1.16 - 1.26 )	1.21 ( 82.81 )	1.2 ( 1.15 )	0.26 ( -1.4 )
Vascular disorders	2120	0.93 ( 0.89 - 0.97 )	0.93 ( 9.67 )	0.94 ( 0.9 )	-0.09 ( -1.76 )
Eye disorders	1474	0.85 ( 0.8 - 0.89 )	0.85 ( 39.5 )	0.85 ( 0.81 )	-0.23 ( -1.9 )
Renal and urinary disorders	1436	1.16 ( 1.1 - 1.22 )	1.16 ( 30.73 )	1.15 ( 1.09 )	0.21 ( -1.46 )
Blood and lymphatic system disorders	1127	0.84 ( 0.79 - 0.89 )	0.84 ( 34.43 )	0.84 ( 0.79 )	-0.25 ( -1.91 )
Immune system disorders	1062	0.99 ( 0.93 - 1.05 )	0.99 ( 0.09 )	0.99 ( 0.93 )	-0.01 ( -1.68 )
Product issues	871	0.9 ( 0.84 - 0.96 )	0.9 ( 10.03 )	0.9 ( 0.84 )	-0.15 ( -1.82 )
Hepatobiliary disorders	787	1.19 ( 1.11 - 1.28 )	1.19 ( 22.87 )	1.18 ( 1.1 )	0.24 ( -1.42 )
Neoplasms benign, malignant and unspecified (incl cysts and polyps)	583	0.35 ( 0.32 - 0.38 )	0.35 ( 702.84 )	0.36 ( 0.33 )	-1.48 ( -3.15 )
Reproductive system and breast disorders	541	0.77 ( 0.71 - 0.84 )	0.77 ( 35.39 )	0.78 ( 0.71 )	-0.36 ( -2.03 )
Ear and labyrinth disorders	528	1.24 ( 1.14 - 1.35 )	1.24 ( 23.81 )	1.23 ( 1.13 )	0.3 ( -1.37 )
Surgical and medical procedures	419	0.33 ( 0.3 - 0.37 )	0.34 ( 550.74 )	0.34 ( 0.31 )	-1.54 ( -3.21 )
Social circumstances	414	0.98 ( 0.89 - 1.09 )	0.98 ( 0.1 )	0.98 ( 0.89 )	-0.02 ( -1.69 )
Pregnancy, puerperium and perinatal conditions	316	0.99 ( 0.88 - 1.11 )	0.99 ( 0.04 )	0.99 ( 0.88 )	-0.02 ( -1.68 )
Endocrine disorders	187	0.97 ( 0.84 - 1.12 )	0.97 ( 0.15 )	0.97 ( 0.84 )	-0.04 ( -1.71 )
Congenital, familial and genetic disorders	169	1.28 ( 1.1 - 1.5 )	1.28 ( 10.21 )	1.27 ( 1.09 )	0.35 ( -1.32 )

Nervous-system and blood/lymphatic-system SOCs were selected for focused descriptive analyses because they contained recurrent reports and signals across the databases. This prioritization does not imply that the selected agents cause these events, because spontaneous-reporting data cannot establish incidence or causality and are susceptible to reporting bias, confounding by indication, and concomitant medication effects (22, 49).

#### 3.2.3 Signal detection at the PT level

The top 30 PTs were selected the top 30PTs within the SOC of Nervous system disorders and Blood and lymphatic system disorders from the FAERS, JADER, and CVARD databases (**Tables 6**–**11**).

**Table 6:** The signal strength of nervous system disorders AERs for GSK 3β inhibitors at the PT level in the FAERS database (TOP 30).

PT	Case number	ROR (95%Cl)	PRR (chi-square value)	EBGM (EBGM05)	IC (IC025)
Dizziness	6167	1.12 ( 1.1 - 1.15 )	1.12 ( 82.1 )	1.12 ( 1.1 )	0.16 ( 0.13 )
Somnolence	5552	2.56 ( 2.49 - 2.63 )	2.55 ( 5066.33 )	2.5 ( 2.44 )	1.32 ( 1.28 )
Headache	5048	0.72 ( 0.7 - 0.74 )	0.73 ( 524.79 )	0.73 ( 0.71 )	-0.46 ( -0.5 )
Tremor	2933	1.59 ( 1.53 - 1.65 )	1.59 ( 627.82 )	1.58 ( 1.53 )	0.66 ( 0.6 )
Loss of consciousness	2092	1.48 ( 1.42 - 1.54 )	1.48 ( 316.92 )	1.47 ( 1.42 )	0.55 ( 0.49 )
Seizure	2083	1.73 ( 1.65 - 1.8 )	1.73 ( 622.83 )	1.71 ( 1.65 )	0.77 ( 0.71 )
Coma	1781	3.46 ( 3.3 - 3.63 )	3.46 ( 2986.53 )	3.36 ( 3.23 )	1.75 ( 1.68 )
Depressed level of consciousness	1618	3.8 ( 3.61 - 3.99 )	3.79 ( 3178.85 )	3.67 ( 3.52 )	1.87 ( 1.8 )
Dysarthria	1546	3.82 ( 3.63 - 4.02 )	3.81 ( 3060.22 )	3.68 ( 3.53 )	1.88 ( 1.81 )
Sedation	1518	5.97 ( 5.67 - 6.29 )	5.96 ( 5842.17 )	5.62 ( 5.38 )	2.49 ( 2.41 )
Neuroleptic malignant syndrome	1425	12.42 ( 11.75 - 13.13 )	12.4 ( 12944.73 )	10.88 ( 10.38 )	3.44 ( 3.36 )
Syncope	1289	1.16 ( 1.09 - 1.22 )	1.16 ( 26.58 )	1.15 ( 1.1 )	0.21 ( 0.12 )
Memoryimpairment	1160	0.75 ( 0.71 - 0.8 )	0.75 ( 92.8 )	0.76 ( 0.72 )	-0.4 ( -0.49 )
Disturbance in attention	1116	1.83 ( 1.73 - 1.95 )	1.83 ( 413.09 )	1.81 ( 1.73 )	0.86 ( 0.77 )
Lethargy	1096	1.72 ( 1.62 - 1.83 )	1.72 ( 324.25 )	1.71 ( 1.62 )	0.77 ( 0.68 )
Epilepsy	1049	3.27 ( 3.07 - 3.48 )	3.27 ( 1585.34 )	3.18 ( 3.02 )	1.67 ( 1.58 )
Speech disorder	1044	1.8 ( 1.69 - 1.91 )	1.8 ( 360.93 )	1.78 ( 1.69 )	0.83 ( 0.74 )
Dysgeusia	950	1.12 ( 1.05 - 1.19 )	1.12 ( 11.24 )	1.11 ( 1.06 )	0.16 ( 0.06 )
Paraesthesia	947	0.53 ( 0.5 - 0.57 )	0.53 ( 390.39 )	0.53 ( 0.51 )	-0.9 ( -1 )
Hypoaesthesia	920	0.54 ( 0.51 - 0.58 )	0.55 ( 347.68 )	0.55 ( 0.52 )	-0.87 ( -0.96 )
Cerebrovascular accident	911	0.47 ( 0.44 - 0.5 )	0.47 ( 538.35 )	0.47 ( 0.45 )	-1.08 ( -1.17 )
Cognitive disorder	886	1.75 ( 1.64 - 1.87 )	1.75 ( 280.26 )	1.74 ( 1.64 )	0.8 ( 0.7 )
Extrapyramidal disorder	862	3.02 ( 2.82 - 3.24 )	3.02 ( 1123.48 )	2.95 ( 2.78 )	1.56 ( 1.46 )
Dyskinesia	790	1.76 ( 1.64 - 1.89 )	1.76 ( 254.62 )	1.75 ( 1.65 )	0.8 ( 0.7 )
Post-Injection delirium sedation syndrome	760	1137.57 ( 863.17 - 1499.22 )	1136.27 ( 57188.34 )	76.31 ( 60.57 )	6.25 ( 6.11 )
Balance disorder	749	0.77 ( 0.72 - 0.83 )	0.77 ( 51 )	0.77 ( 0.73 )	-0.37 ( -0.48 )
Unresponsive to stimuli	742	2.7 ( 2.51 - 2.9 )	2.69 ( 765.3 )	2.64 ( 2.48 )	1.4 ( 1.29 )
Migraine	736	0.72 ( 0.67 - 0.77 )	0.72 ( 81.47 )	0.72 ( 0.68 )	-0.47 ( -0.58 )
Altered state of consciousness	705	3.14 ( 2.91 - 3.39 )	3.14 ( 988.82 )	3.06 ( 2.87 )	1.61 ( 1.5 )
Parkinsonism	678	7 ( 6.47 - 7.57 )	6.99 ( 3204.42 )	6.51 ( 6.1 )	2.7 ( 2.59 )

**Table 7:** The signal strength of blood and lymphatic system disorders AERs for GSK 3βinhibitors at the PT level in the FAERS database (TOP 30).

PT	Case number	ROR (95%Cl)	PRR (chi-square value)	EBGM (EBGM05)	IC (IC025)
Anaemia	1469	0.68 ( 0.65 - 0.72 )	0.68 ( 213.11 )	0.69 ( 0.66 )	-0.54 ( -0.62 )
Neutropenia	1114	0.74 ( 0.7 - 0.79 )	0.74 ( 99.7 )	0.74 ( 0.71 )	-0.43 ( -0.51 )
Thrombocytopenia	1082	0.88 ( 0.83 - 0.94 )	0.88 ( 17.17 )	0.88 ( 0.84 )	-0.18 ( -0.27 )
Leukopenia	585	1.06 ( 0.98 - 1.15 )	1.06 ( 2 )	1.06 ( 0.99 )	0.08 ( -0.04 )
Agranulocytosis	491	2.54 ( 2.32 - 2.78 )	2.54 ( 443.87 )	2.49 ( 2.31 )	1.32 ( 1.18 )
Leukocytosis	436	2.23 ( 2.03 - 2.45 )	2.23 ( 287.32 )	2.2 ( 2.03 )	1.13 ( 0.99 )
Pancytopenia	391	0.64 ( 0.58 - 0.71 )	0.64 ( 78.25 )	0.64 ( 0.59 )	-0.64 ( -0.78 )
Eosinophilia	382	2.04 ( 1.84 - 2.26 )	2.04 ( 197.26 )	2.01 ( 1.85 )	1.01 ( 0.86 )
Methaemoglobinaemia	358	18.88 ( 16.82 - 21.18 )	18.87 ( 4909.96 )	15.48 ( 14.06 )	3.95 ( 3.79 )
Coagulopathy	310	1.67 ( 1.5 - 1.87 )	1.67 ( 82.5 )	1.66 ( 1.51 )	0.73 ( 0.57 )
Lymphadenopathy	303	0.77 ( 0.69 - 0.86 )	0.77 ( 20.92 )	0.77 ( 0.7 )	-0.38 ( -0.54 )
Disseminated intravascular coagulation	162	1.01 ( 0.86 - 1.18 )	1.01 ( 0.01 )	1.01 ( 0.89 )	0.01 ( -0.21 )
Iron deficiency anaemia	147	1.47 ( 1.25 - 1.73 )	1.47 ( 21.92 )	1.46 ( 1.28 )	0.55 ( 0.31 )
Lymphopenia	94	0.61 ( 0.49 - 0.74 )	0.61 ( 24.05 )	0.61 ( 0.51 )	-0.72 ( -1.01 )
Bone marrow failure	88	0.37 ( 0.3 - 0.45 )	0.37 ( 94.5 )	0.37 ( 0.31 )	-1.43 ( -1.73 )
Splenomegaly	88	0.67 ( 0.54 - 0.82 )	0.67 ( 14.67 )	0.67 ( 0.56 )	-0.58 ( -0.89 )
Febrile neutropenia	87	0.12 ( 0.1 - 0.15 )	0.12 ( 565.81 )	0.12 ( 0.1 )	-3.05 ( -3.36 )
Neutrophilia	85	1.4 ( 1.13 - 1.74 )	1.4 ( 9.63 )	1.4 ( 1.17 )	0.48 ( 0.17 )
Granulocytopenia	82	1.23 ( 0.99 - 1.53 )	1.23 ( 3.56 )	1.23 ( 1.02 )	0.3 ( -0.02 )
Haemolytic anaemia	82	0.82 ( 0.66 - 1.02 )	0.82 ( 3.22 )	0.82 ( 0.68 )	-0.28 ( -0.6 )
Blood disorder	80	0.89 ( 0.71 - 1.11 )	0.89 ( 1.08 )	0.89 ( 0.74 )	-0.17 ( -0.49 )
Haemolysis	74	0.86 ( 0.68 - 1.08 )	0.86 ( 1.64 )	0.86 ( 0.71 )	-0.21 ( -0.55 )
Thrombotic microangiopathy	56	0.55 ( 0.42 - 0.71 )	0.55 ( 20.87 )	0.55 ( 0.44 )	-0.86 ( -1.25 )
Thrombotic thrombocytopenic purpura	51	1.29 ( 0.98 - 1.7 )	1.29 ( 3.22 )	1.28 ( 1.02 )	0.36 ( -0.04 )
Increased tendency to bruise	50	0.47 ( 0.36 - 0.62 )	0.47 ( 29.33 )	0.48 ( 0.38 )	-1.07 ( -1.48 )
Microcytic anaemia	48	2.07 ( 1.56 - 2.76 )	2.07 ( 25.93 )	2.04 ( 1.61 )	1.03 ( 0.61 )
Myelosuppression	47	0.18 ( 0.13 - 0.24 )	0.18 ( 178.19 )	0.18 ( 0.14 )	-2.48 ( -2.89 )
Normochromic normocytic anaemia	41	1.2 ( 0.88 - 1.63 )	1.2 ( 1.36 )	1.2 ( 0.93 )	0.26 ( -0.19 )
Aplastic anaemia	40	0.7 ( 0.51 - 0.95 )	0.7 ( 5.15 )	0.7 ( 0.54 )	-0.51 ( -0.96 )
Haemorrhagic diathesis	39	0.95 ( 0.69 - 1.3 )	0.95 ( 0.1 )	0.95 ( 0.73 )	-0.07 ( -0.53 )

**Table 8:** The signal strength of nervous system disorders AERs for GSK 3βinhibitors at the PT level in the JADER database (TOP 30).

PT	Case number	ROR (95%Cl)	PRR (chi-square value)	EBGM (EBGM05)	IC (IC025)
Neuroleptic malignant syndrome	756	17.42 ( 16.08 - 18.88 )	16.82 ( 9216.37 )	13.93 ( 12.86 )	3.8 ( 2.13 )
Altered state of consciousness	297	2.92 ( 2.6 - 3.29 )	2.9 ( 357.06 )	2.83 ( 2.52 )	1.5 ( -0.17 )
Seizure	296	2.71 ( 2.41 - 3.05 )	2.69 ( 304.43 )	2.63 ( 2.34 )	1.39 ( -0.27 )
Epilepsy	148	3.78 ( 3.21 - 4.47 )	3.76 ( 286.63 )	3.63 ( 3.08 )	1.86 ( 0.19 )
Depressed level of consciousness	120	2.13 ( 1.78 - 2.56 )	2.12 ( 69.59 )	2.09 ( 1.74 )	1.07 ( -0.6 )
Loss of consciousness	104	1.05 ( 0.86 - 1.27 )	1.05 ( 0.22 )	1.05 ( 0.86 )	0.07 ( -1.6 )
Dystonia	103	10.78 ( 8.77 - 13.26 )	10.74 ( 796.21 )	9.52 ( 7.74 )	3.25 ( 1.58 )
Parkinsonism	102	8.78 ( 7.15 - 10.78 )	8.74 ( 626.96 )	7.94 ( 6.46 )	2.99 ( 1.32 )
Somnolence	89	2.99 ( 2.42 - 3.69 )	2.98 ( 112.64 )	2.9 ( 2.35 )	1.54 ( -0.13 )
Tardive dyskinesia	80	9.64 ( 7.63 - 12.16 )	9.6 ( 546.93 )	8.63 ( 6.83 )	3.11 ( 1.44 )
Extrapyramidal disorder	75	11.37 ( 8.92 - 14.5 )	11.33 ( 614.32 )	9.98 ( 7.83 )	3.32 ( 1.65 )
Dyskinesia	62	3.95 ( 3.06 - 5.1 )	3.94 ( 129.38 )	3.79 ( 2.94 )	1.92 ( 0.26 )
Dizziness	61	0.81 ( 0.63 - 1.04 )	0.81 ( 2.64 )	0.81 ( 0.63 )	-0.3 ( -1.96 )
Tremor	61	2.54 ( 1.97 - 3.28 )	2.54 ( 54.97 )	2.49 ( 1.93 )	1.31 ( -0.35 )
Generalised tonic-clonic seizure	60	9.05 ( 6.92 - 11.84 )	9.03 ( 382.68 )	8.17 ( 6.25 )	3.03 ( 1.36 )
Serotonin syndrome	53	4.23 ( 3.21 - 5.58 )	4.22 ( 123.4 )	4.05 ( 3.07 )	2.02 ( 0.35 )
Akathisia	50	9.67 ( 7.2 - 12.98 )	9.65 ( 343.61 )	8.66 ( 6.45 )	3.12 ( 1.44 )
Status epilepticus	47	5.55 ( 4.13 - 7.47 )	5.54 ( 163.09 )	5.23 ( 3.89 )	2.39 ( 0.72 )
Intraventricular haemorrhage	39	11.44 ( 8.17 - 16.03 )	11.42 ( 321.98 )	10.05 ( 7.17 )	3.33 ( 1.65 )
Coma	37	5.27 ( 3.78 - 7.36 )	5.26 ( 119.46 )	4.98 ( 3.57 )	2.32 ( 0.65 )
Dementia	34	1.97 ( 1.4 - 2.76 )	1.96 ( 15.68 )	1.94 ( 1.38 )	0.96 ( -0.71 )
Encephalopathy	33	0.98 ( 0.7 - 1.38 )	0.98 ( 0.01 )	0.98 ( 0.7 )	-0.03 ( -1.69 )
Headache	33	0.53 ( 0.38 - 0.75 )	0.53 ( 13.44 )	0.54 ( 0.38 )	-0.9 ( -2.57 )
Cognitive disorder	30	4.09 ( 2.83 - 5.9 )	4.08 ( 66.25 )	3.92 ( 2.72 )	1.97 ( 0.3 )
Hyperammonaemic encephalopathy	28	55.52 ( 34.07 - 90.48 )	55.45 ( 861.97 )	32.35 ( 19.85 )	5.02 ( 3.31 )
Hypoxic-ischaemic encephalopathy	27	3.87 ( 2.63 - 5.69 )	3.86 ( 54.5 )	3.72 ( 2.53 )	1.9 ( 0.23 )
Cerebral infarction	22	0.16 ( 0.1 - 0.24 )	0.16 ( 99.83 )	0.16 ( 0.1 )	-2.65 ( -4.32 )
Motor dysfunction	21	9.82 ( 6.23 - 15.48 )	9.82 ( 147.09 )	8.8 ( 5.58 )	3.14 ( 1.46 )
Cerebral haemorrhage	20	0.21 ( 0.14 - 0.33 )	0.21 ( 58.3 )	0.22 ( 0.14 )	-2.22 ( -3.88 )
Stupor	17	8.95 ( 5.41 - 14.8 )	8.95 ( 107.24 )	8.1 ( 4.9 )	3.02 ( 1.34 )

**Table 9:** The signal strength of blood and lymphatic system disorders AERs for GSK 3βinhibitors at the PT level in the JADER database (TOP 30).

PT	Case number	ROR (95%Cl)	PRR (chi-square value)	EBGM (EBGM05)	IC (IC025)
Thrombocytopenia	95	0.9 ( 0.73 - 1.1 )	0.9 ( 1.14 )	0.9 ( 0.73 )	-0.16 ( -1.82 )
Pancytopenia	83	0.81 ( 0.66 - 1.01 )	0.82 ( 3.46 )	0.82 ( 0.66 )	-0.29 ( -1.96 )
Agranulocytosis	56	0.93 ( 0.72 - 1.22 )	0.93 ( 0.26 )	0.93 ( 0.72 )	-0.1 ( -1.76 )
Disseminated intravascular coagulation	49	0.81 ( 0.61 - 1.08 )	0.81 ( 2.13 )	0.81 ( 0.61 )	-0.3 ( -1.96 )
Anaemia	46	0.22 ( 0.16 - 0.29 )	0.22 ( 130.3 )	0.22 ( 0.16 )	-2.18 ( -3.85 )
Neutropenia	42	0.21 ( 0.16 - 0.29 )	0.21 ( 123.28 )	0.21 ( 0.16 )	-2.22 ( -3.88 )
Leukopenia	41	0.58 ( 0.43 - 0.79 )	0.58 ( 12.31 )	0.58 ( 0.43 )	-0.77 ( -2.44 )
Aplastic anaemia	21	2.29 ( 1.48 - 3.54 )	2.29 ( 14.82 )	2.25 ( 1.46 )	1.17 ( -0.5 )
Aplasia pure red cell	19	2.2 ( 1.39 - 3.47 )	2.2 ( 12.08 )	2.17 ( 1.37 )	1.11 ( -0.55 )
Granulocytopenia	18	1.63 ( 1.02 - 2.6 )	1.63 ( 4.25 )	1.61 ( 1.01 )	0.69 ( -0.98 )
Myelosuppression	18	0.17 ( 0.11 - 0.27 )	0.17 ( 71.95 )	0.17 ( 0.11 )	-2.52 ( -4.19 )
Lymphadenopathy	14	1.46 ( 0.86 - 2.48 )	1.46 ( 2.01 )	1.45 ( 0.86 )	0.54 ( -1.13 )
Haemolytic anaemia	13	0.89 ( 0.51 - 1.53 )	0.89 ( 0.19 )	0.89 ( 0.51 )	-0.17 ( -1.84 )
Thrombotic microangiopathy	13	0.73 ( 0.42 - 1.26 )	0.73 ( 1.32 )	0.73 ( 0.42 )	-0.45 ( -2.12 )
Eosinophilia	12	1.67 ( 0.94 - 2.96 )	1.67 ( 3.17 )	1.66 ( 0.94 )	0.73 ( -0.94 )
Immune thrombocytopenia	11	0.56 ( 0.31 - 1.01 )	0.56 ( 3.78 )	0.56 ( 0.31 )	-0.83 ( -2.5 )
Febrile neutropenia	11	0.06 ( 0.03 - 0.11 )	0.06 ( 159.26 )	0.06 ( 0.03 )	-4.01 ( -5.68 )
Methaemoglobinaemia	10	6.38 ( 3.34 - 12.17 )	6.38 ( 41.8 )	5.96 ( 3.12 )	2.57 ( 0.89 )
Cytopenia	5	0.27 ( 0.11 - 0.66 )	0.27 ( 9.55 )	0.28 ( 0.12 )	-1.85 ( -3.52 )
Autoimmune haemolytic anaemia	4	0.73 ( 0.27 - 1.94 )	0.73 ( 0.41 )	0.73 ( 0.27 )	-0.46 ( -2.13 )
Haemorrhagic diathesis	4	0.43 ( 0.16 - 1.16 )	0.43 ( 2.96 )	0.44 ( 0.16 )	-1.2 ( -2.87 )
Iron deficiency anaemia	4	0.99 ( 0.37 - 2.65 )	0.99 ( 0 )	0.99 ( 0.37 )	-0.02 ( -1.69 )
Thrombocytopenic purpura	3	0.4 ( 0.13 - 1.25 )	0.4 ( 2.66 )	0.4 ( 0.13 )	-1.3 ( -2.97 )
Anaemia macrocytic	3	1.93 ( 0.61 - 6.07 )	1.93 ( 1.31 )	1.91 ( 0.61 )	0.93 ( -0.76 )
Bone marrow disorder	2	2.28 ( 0.56 - 9.31 )	2.28 ( 1.4 )	2.24 ( 0.55 )	1.17 ( -0.54 )
Anaemia folate deficiency	2	3.42 ( 0.83 - 14.11 )	3.42 ( 3.28 )	3.32 ( 0.8 )	1.73 ( 0.01 )
Bone marrow failure	2	0.44 ( 0.11 - 1.78 )	0.44 ( 1.39 )	0.45 ( 0.11 )	-1.16 ( -2.83 )
Anaemia megaloblastic	2	2.32 ( 0.57 - 9.46 )	2.32 ( 1.45 )	2.28 ( 0.56 )	1.19 ( -0.52 )
Normochromic normocytic anaemia	2	7.92 ( 1.85 - 34.01 )	7.92 ( 10.94 )	7.26 ( 1.69 )	2.86 ( 1.08 )
Thrombotic thrombocytopenic purpura	2	0.32 ( 0.08 - 1.29 )	0.32 ( 2.85 )	0.32 ( 0.08 )	-1.62 ( -3.3 )

**Table 10:** The signal strength of nervous system disorders AERs for GSK 3βinhibitors at the PT level in the CVARD database (TOP 30).

PT	Case number	ROR (95%Cl)	PRR (chi-square value)	EBGM (EBGM05)	IC (IC025)
Dizziness	1330	1.44 ( 1.36 - 1.52 )	1.43 ( 168.97 )	1.42 ( 1.34 )	0.5 ( -1.16 )
Headache	941	0.81 ( 0.76 - 0.86 )	0.81 ( 41.57 )	0.81 ( 0.76 )	-0.3 ( -1.96 )
Somnolence	660	2.36 ( 2.18 - 2.55 )	2.35 ( 478.99 )	2.26 ( 2.09 )	1.18 ( -0.49 )
Seizure	556	2.28 ( 2.09 - 2.49 )	2.28 ( 372.28 )	2.19 ( 2.01 )	1.13 ( -0.53 )
Tremor	533	1.92 ( 1.76 - 2.1 )	1.92 ( 221.56 )	1.87 ( 1.71 )	0.9 ( -0.77 )
Sedation	368	12.97 ( 11.49 - 14.64 )	12.93 ( 2887.94 )	9.5 ( 8.42 )	3.25 ( 1.58 )
Loss of consciousness	300	1.49 ( 1.33 - 1.67 )	1.49 ( 45.75 )	1.46 ( 1.3 )	0.55 ( -1.12 )
Depressed level of consciousness	280	4.72 ( 4.16 - 5.35 )	4.71 ( 713.76 )	4.23 ( 3.73 )	2.08 ( 0.42 )
Dyskinesia	240	6.14 ( 5.35 - 7.05 )	6.13 ( 865.47 )	5.31 ( 4.62 )	2.41 ( 0.74 )
Hypoaesthesia	231	0.7 ( 0.62 - 0.8 )	0.7 ( 28.48 )	0.71 ( 0.62 )	-0.5 ( -2.16 )
Lethargy	210	2.53 ( 2.19 - 2.91 )	2.52 ( 179.17 )	2.41 ( 2.1 )	1.27 ( -0.4 )
Dystonia	207	10.13 ( 8.67 - 11.85 )	10.11 ( 1293.23 )	7.93 ( 6.78 )	2.99 ( 1.32 )
Paraesthesia	202	0.72 ( 0.63 - 0.83 )	0.72 ( 21.12 )	0.73 ( 0.63 )	-0.46 ( -2.12 )
Syncope	185	1.11 ( 0.96 - 1.29 )	1.11 ( 2.03 )	1.11 ( 0.96 )	0.15 ( -1.52 )
Balance disorder	178	1.36 ( 1.17 - 1.58 )	1.36 ( 16.31 )	1.35 ( 1.16 )	0.43 ( -1.24 )
Cerebrovascular accident	177	0.91 ( 0.78 - 1.06 )	0.91 ( 1.52 )	0.91 ( 0.79 )	-0.13 ( -1.8 )
Cognitive disorder	150	3.01 ( 2.55 - 3.56 )	3.01 ( 184.14 )	2.84 ( 2.4 )	1.5 ( -0.16 )
Anosognosia	146	234.69 ( 147.07 - 374.51 )	234.36 ( 4087.54 )	29.12 ( 18.25 )	4.86 ( 3.18 )
Neuroleptic malignant syndrome	136	7.47 ( 6.2 - 9.01 )	7.46 ( 617.79 )	6.24 ( 5.18 )	2.64 ( 0.97 )
Coma	127	3.52 ( 2.93 - 4.23 )	3.52 ( 206.65 )	3.27 ( 2.72 )	1.71 ( 0.04 )
Migraine	127	0.65 ( 0.54 - 0.77 )	0.65 ( 23.94 )	0.65 ( 0.55 )	-0.61 ( -2.28 )
Generalised tonic-clonic seizure	119	3.12 ( 2.59 - 3.77 )	3.12 ( 156.17 )	2.93 ( 2.43 )	1.55 ( -0.12 )
Akathisia	117	9.17 ( 7.47 - 11.26 )	9.16 ( 661.9 )	7.35 ( 5.98 )	2.88 ( 1.21 )
Memory impairment	115	0.97 ( 0.8 - 1.16 )	0.97 ( 0.13 )	0.97 ( 0.8 )	-0.05 ( -1.71 )
Dysarthria	114	2.25 ( 1.86 - 2.72 )	2.25 ( 74.09 )	2.17 ( 1.79 )	1.12 ( -0.55 )
Speech disorder	106	1.32 ( 1.09 - 1.61 )	1.32 ( 8.04 )	1.31 ( 1.08 )	0.39 ( -1.28 )
Parkinsonism	101	9.78 ( 7.82 - 12.21 )	9.77 ( 609.46 )	7.72 ( 6.18 )	2.95 ( 1.28 )
Disturbance in attention	95	1.14 ( 0.93 - 1.4 )	1.14 ( 1.52 )	1.13 ( 0.92 )	0.18 ( -1.49 )
Burning sensation	93	0.69 ( 0.56 - 0.85 )	0.69 ( 12.63 )	0.7 ( 0.57 )	-0.52 ( -2.19 )
Amnesia	89	0.98 ( 0.8 - 1.21 )	0.98 ( 0.03 )	0.98 ( 0.8 )	-0.03 ( -1.69 )

**Table 11:** The signal strength of blood and lymphatic system disorders AERs for GSK 3βinhibitors at the PT level in the CVARD database (TOP 30).

PT	Case number	ROR (95%Cl)	PRR (chi-square value)	EBGM (EBGM05)	IC (IC025)
Anaemia	169	0.94 ( 0.8 - 1.09 )	0.94 ( 0.72 )	0.94 ( 0.8 )	-0.09 ( -1.76 )
Thrombocytopenia	141	1.12 ( 0.95 - 1.33 )	1.12 ( 1.84 )	1.12 ( 0.95 )	0.16 ( -1.5 )
Leukopenia	122	1.44 ( 1.2 - 1.73 )	1.44 ( 15.96 )	1.43 ( 1.19 )	0.51 ( -1.16 )
Neutropenia	122	0.55 ( 0.46 - 0.66 )	0.55 ( 44.36 )	0.56 ( 0.47 )	-0.84 ( -2.51 )
Leukocytosis	64	1.92 ( 1.49 - 2.47 )	1.92 ( 26.49 )	1.87 ( 1.45 )	0.9 ( -0.77 )
Lymphadenopathy	53	0.74 ( 0.56 - 0.97 )	0.74 ( 4.94 )	0.74 ( 0.56 )	-0.43 ( -2.1 )
Agranulocytosis	45	1.66 ( 1.23 - 2.24 )	1.66 ( 11.19 )	1.63 ( 1.21 )	0.7 ( -0.97 )
Methaemoglobinaemia	44	13.99 ( 9.82 - 19.94 )	13.99 ( 369.55 )	10.05 ( 7.05 )	3.33 ( 1.65 )
Pancytopenia	30	0.58 ( 0.4 - 0.83 )	0.58 ( 9.08 )	0.59 ( 0.41 )	-0.77 ( -2.44 )
Eosinophilia	29	1.3 ( 0.9 - 1.89 )	1.3 ( 1.95 )	1.29 ( 0.89 )	0.37 ( -1.3 )
Lymphadenopathy mediastinal	29	13.3 ( 8.63 - 20.51 )	13.3 ( 233.26 )	9.7 ( 6.29 )	3.28 ( 1.59 )
Neutrophilia	18	1.58 ( 0.98 - 2.53 )	1.58 ( 3.64 )	1.55 ( 0.97 )	0.63 ( -1.04 )
Anaemia macrocytic	15	5.8 ( 3.35 - 10.06 )	5.8 ( 50.49 )	5.07 ( 2.92 )	2.34 ( 0.65 )
Coagulopathy	13	0.95 ( 0.55 - 1.65 )	0.95 ( 0.04 )	0.95 ( 0.55 )	-0.07 ( -1.75 )
Granulocytopenia	13	0.88 ( 0.51 - 1.53 )	0.88 ( 0.21 )	0.88 ( 0.51 )	-0.18 ( -1.85 )
Iron deficiency anaemia	12	1.22 ( 0.68 - 2.16 )	1.22 ( 0.44 )	1.21 ( 0.68 )	0.27 ( -1.4 )
White blood cell disorder	11	1.27 ( 0.7 - 2.32 )	1.27 ( 0.61 )	1.26 ( 0.69 )	0.33 ( -1.34 )
Febrile neutropenia	10	0.09 ( 0.05 - 0.16 )	0.09 ( 96.52 )	0.09 ( 0.05 )	-3.49 ( -5.16 )
Splenomegaly	9	0.53 ( 0.27 - 1.03 )	0.53 ( 3.67 )	0.54 ( 0.28 )	-0.89 ( -2.56 )
Haemolytic anaemia	8	0.39 ( 0.19 - 0.78 )	0.39 ( 7.69 )	0.39 ( 0.2 )	-1.34 ( -3.01 )
Lymphopenia	8	0.59 ( 0.29 - 1.18 )	0.59 ( 2.29 )	0.59 ( 0.3 )	-0.75 ( -2.42 )
Immune thrombocytopenia	8	0.58 ( 0.29 - 1.17 )	0.58 ( 2.35 )	0.59 ( 0.29 )	-0.76 ( -2.43 )
Increased tendency to bruise	8	0.69 ( 0.34 - 1.39 )	0.69 ( 1.09 )	0.7 ( 0.35 )	-0.52 ( -2.19 )
Macrocytosis	7	2.44 ( 1.13 - 5.27 )	2.44 ( 5.54 )	2.34 ( 1.09 )	1.23 ( -0.46 )
Thrombocytosis	7	1.64 ( 0.77 - 3.51 )	1.64 ( 1.66 )	1.61 ( 0.75 )	0.69 ( -1 )
Normocytic anaemia	7	3.57 ( 1.63 - 7.79 )	3.57 ( 11.64 )	3.31 ( 1.52 )	1.73 ( 0.03 )
Blood disorder	7	0.72 ( 0.34 - 1.51 )	0.72 ( 0.77 )	0.72 ( 0.34 )	-0.47 ( -2.14 )
Aplastic anaemia	6	0.66 ( 0.3 - 1.49 )	0.66 ( 1 )	0.67 ( 0.3 )	-0.58 ( -2.25 )
Platelet production decreased	6	3.32 ( 1.43 - 7.7 )	3.32 ( 8.82 )	3.1 ( 1.34 )	1.63 ( -0.07 )
Lymph node calcification	6	7.71 ( 3.16 - 18.78 )	7.7 ( 28.23 )	6.41 ( 2.63 )	2.68 ( 0.94 )

In the FAERS database, Valproate was associated with 14,999 cases (19.59%) of adverse reactions in the Nervous system disorders, Olanzapine with 22,385 cases (17.42%), and Lithium with 4969 cases (19.12%) (**Supplementary Figures 7A, B**). In the Blood and lymphatic system disorders, Valproate was associated with 1894 cases (2.47%) of adverse reactions, Olanzapine with 2441 cases (1.90%), and Ibuprofen with 4121 cases (1.25%) (**Supplementary Figures 7C, D**).

Among all drugs, 1225 PTs (14.1%) were positive in all four algorithms (ROR, PRR, EBGM, BCPNN) (**Supplementary Figure 8A**).

In the Nervous system disorder of the adverse reactions with the largest number of reports were Dizziness(n=6167), Somnolence(n=5552), and Headache(n=5048) (**Figure 6A**). And in the Blood and lymphatic system disorders of the adverse reactions with the largest number of reports were Anemia (n=1469), Neutropenia(n=1114), and Thrombocytopenia(n=585) (**Figure 6B**).

**Figure 6:**
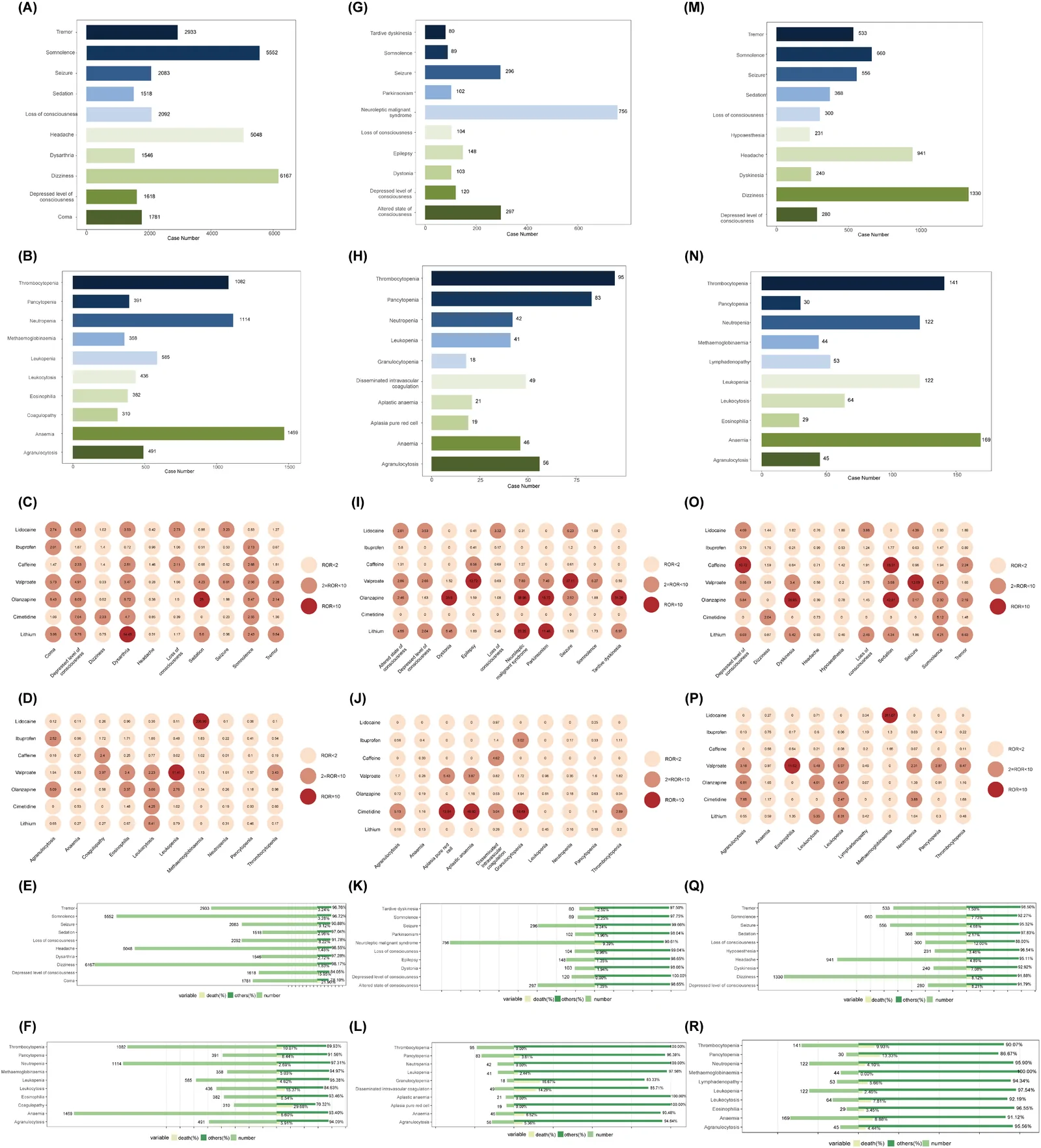
Signals detection at the top 10 PTs of target SOCs **(A)** shows the top ten PTs with the highest number of nervous system disorders SOC in FAERS database **(B)** shows the top ten PTs with the highest number of blood and lymphatic system disorders SOC in FAERS database **(C)**. The heatmap shows the ROR value of the top ten PTs of nervous system disorders SOC in FAERS database **(D)**. The heatmap shows the ROR value of the top ten PTs of blood and lymphatic system disorders SOC in FAERS database **(E)** shows the fatality rate and number of reports for top ten PTs of nervous system disorders SOC in FAERS database **(F)** shows the fatality rate and number of reports for top ten PTs of blood and lymphatic system disorders SOC in FAERS database **(G)** shows the top ten PTs with the highest number of nervous system disorders SOC in JADER database **(H)** shows the top ten PTs with the highest number of blood and lymphatic system disorders SOC in JADER database **(I)**. The heatmap shows the ROR value of the top ten PTs of nervous system disorders SOC in JADER database **(J)**. The heatmap shows the ROR value of the top ten PTs of blood and lymphatic system disorders SOC in JADER database **(K)** shows the fatality rate and number of reports for top ten PTs of nervous system disorders SOC in JADER database **(L)** shows the fatality rate and number of reports for top ten PTs of blood and lymphatic system disorders SOC in JADER database **(M)** shows the top ten PTs with the highest number of nervous system disorders SOC in CVARD database **(N)** shows the top ten PTs with the highest number of blood and lymphatic system disorders SOC in CVARD database **(O)**. The heatmap shows the ROR value of the top ten PTs of nervous system disorders SOC in CVARD database **(P)**. The heatmap shows the ROR value of the top ten PTs of blood and lymphatic system disorders SOC in CVARD database **(Q)** shows the fatality rate and number of reports for top ten PTs of nervous system disorders SOC in CVARD database **(R)** shows the fatality rate and number of reports for top ten PTs of blood and lymphatic system disorders SOC in CVARD database.

Olanzapine was most associated with sedation (206.96), while Lidocaine was most associated with methaelobinemia (ROR = 206.96) (**Figures 6C, D**; **Supplementary Figures 9A, B**).

In the adverse reactions associated with GSK-3β inhibitors, coma has the highest mortality rate (21.90%) in nervous system disorders (**Figure 6E**), while coagulopathy has the highest mortality rate (29.68%) in blood and lymphatic system disorders (**Figure 6F**).

In the JADER database, Olanzapine was associated with 1446 cases (25.39%) of adverse reactions in the Nervous system disorders, Valproate with 1213 cases (16.24%), and Lithium with 370 cases (15.11%) (**Supplementary Figures 7E, F**). In the Blood and lymphatic system disorders, Cimetidine was associated with 40 cases (11.87%) of adverse reactions Valproate with 381 cases (5.10%), and Ibuprofen with 37 cases (3.04%) (**Supplementary Figures 7G, H**).

Among all drugs, 215 PTs (11.7%) were positive in all four algorithms (ROR, PRR, EBGM, BCPNN) (**Supplementary Figure 8B**).

In the Nervous system disorder of the adverse reactions with the largest number of reports were Neuroleptic malignant syndrome(n=756), Altered state of consciousness(n=297), and Seizure(n=296) (**Figure 6G**). And in the Blood and lymphatic system disorders of the adverse reactions with the largest number of reports were Thrombocytopenia(n=95), Pancytopenia(n=83), and Agranulocytosis((n=56) (**Figure 6H**).

Olanzapine was most associated with neuroleptic malignant syndrome (ROR = 38.96), while Cimetidine was most associated with aplastic anemia (ROR = 46.92) (**Figures 6I, J**; **Supplementary Figures 9C, D**).

In the adverse reactions associated with GSK-3β inhibitors, Neuroleptic malignant syndrome has the highest mortality rate (9.39%) in nervous system disorders (**Figure 6K**), while Granulocytopenia has the highest mortality rate (16.67%) in blood and lymphatic system disorders (**Figure 6L**).

In the CVARD database, Valproate was associated with 338 cases (3.57%) of adverse reactions in the Nervous system disorders, Cimetidine with 13 cases (2.66%), and Olanzapine with 369 cases (1.86%) (**Supplementary Figures 7, I, J**). In the Blood and lymphatic system disorders, Lithium was associated with 1091 cases (16.02%) of adverse reactions Olanzapine with 3100 cases (15.62%), and Valproate with 1438 cases (15.21%) (**Supplementary Figures 7K, L**).

Among all drugs, 352 PTs (9.5%) were positive in all four algorithms (ROR, PRR, EBGM, BCPNN) (**Supplementary Figure 8C**).

In the Nervous system disorder of the adverse reactions with the largest number of reports were Dizziness(n=1330), Headache(n=941), and Somnolence(n=660) (**Figure 6M**). And in the Blood and lymphatic system disorders of the adverse reactions with the largest number of reports were Anemia(n=169), Thrombocytopenia(n=141), Neutropenia(n=122) and Leukopenia(n=122) (**Figure 6N**).

Olanzapine was most associated with sedation (ROR = 42.81), while Lidocaine was most associated with methaemoglobinemia (ROR = 311.07) (**Figures 6O, P**; **Supplementary Figures 9E, F**).

In the adverse reactions associated with GSK-3β inhibitors, Loss of consciousness has the highest mortality rate (12.00%) in nervous system disorders (**Figure 6Q**), while Pancytopenia has the highest mortality rate (13.33%) in blood and lymphatic system disorders (**Figure 6R**).

#### 3.2.4 Signal detection at the HLT and HLGT levels

The analysis examined drug adverse reactions at the HLT and HLGT levels and created a Sankey diagram for the top ten PTs in the SOC category of Nervous system disorders and Blood and lymphatic system disorders. The analysis first examined the data at the HLT level and found that in the FAERS database, valproate had the strongest correlation with reticuloendothelial disorder nec in the blood and lymphatic system disorders SOC (**Supplementary Figure 10A**), while valproate had the strongest correlation with intellectual disabilities in the nervous system disorders SOC (**Supplementary Figure 10B**). In the blood and lymphatic system disorders, neutropenias HLT and white blood cell disorders HLGT were reported most frequently (**Supplementary Figure 10C**). In the nervous system disorders, disturbances in consciousness nec HLT and neurological disorders nec HLGT were reported most frequently (**Supplementary Figure 10D**). In the JADER database, cimetidine had the strongest correlation with anemias hemolytic nec in the blood and lymphatic system disorders SOC (**Supplementary Figure 10E**), while ibuprofen had the strongest correlation with vertigos nec in the nervous system disorders SOC (**Supplementary Figure 10F**). In the blood and lymphatic system disorders, marrow depression and hypoplastic anemias HLT and white blood cell disorders HLGT were reported most frequently (**Supplementary Figure 10G**). In the nervous system disorders, disturbances in muscle tone abnormal HLT and neuromuscular disorders HLGT were reported most frequently (**Supplementary Figure 10H**). In the CVARD database, ibuprofen had the strongest correlation with hematological disorders nec in the blood and lymphatic system disorders SOC (**Supplementary Figure 10I**), while valproate had the strongest correlation with seizures (incl subtypes) in the nervous system disorders SOC (**Supplementary Figure 10J**). In the blood and lymphatic system disorders, neutropenias HLT and white blood cell disorders HLGT were reported most frequently (**Supplementary Figure 10K**). In the nervous system disorders, disturbances in disturbances in consciousness nec HLT and neurological disorders HLGT were reported most frequently (**Supplementary Figure 10L**).

#### 3.2.5 Clinical significance analysis

Clinical-priority analysis was conducted clinical priority analysis separately across the different databases. We listed the PT with the top 30 clinical priorities for analysis, with no strong priorities in any of the three databases (**Supplementary Tables 6**-**11**).

#### 3.2.6 TTO analysis

Time to onset (TTO) was examined in FAERS only for the seven analyzed agents: olanzapine, valproate, ibuprofen, caffeine, lithium, lidocaine, and cimetidine. **Supplementary Figure 11A** presents the overall FAERS TTO distributions. Because corresponding onset-date data were unavailable in JADER and CVARD, these results are descriptive and cannot be compared across the three databases.

Subsequently, TTO was analyzed the TTO at the SOC level of the same seven drugs. The TTO cumulative distribution curve shows that among Nervous system disorders and Blood and lymphatic system disorders, the onset of diseases occurs earliest in the Blood and lymphatic system disorders (**Supplementary Figures 11B–I**).

In the FAERS TTO dataset, many reported events occurred within the first month after administration, followed by fewer reports over time; a later increase was observed between 181 and 360 days for some agents (**Supplementary Figure 12**). These reporting-time patterns should be interpreted cautiously because missing or inaccurate dates, treatment interruptions, and reporting practices can influence TTO estimates.

We subsequently analyzed the median and quartiles of adverse reaction induction times across different prioritization levels in the FAERS database. In the Nervous system disorder of the adverse reactions, the median and interquartile range for moderate prioritization was 31 days (3.5–375 days), and for weak prioritization it was 51 days (7–365 days). In the Blood and lymphatic system disorders of the adverse reactions, the median and interquartile range for moderate prioritization was 8 days (3–19 days), and for weak prioritization it was 13 days (4–61 days). A Weibull analysis was also performed a Weibull analysis. The Weibull distribution parameters showed that the shape parameter (β) was less than 1, indicating an early failure TTO distribution pattern in both blood and lymphatic system disorders and nervous system disorders (**Supplementary Tables 12**, **13**).

#### 3.2.7 Gender subgroup analysis

To assess whether gender influences drug adverse reactions, ROR and PRR methods were used the ROR and PRR method to identify PTs with disproportional AEs incidence between males and females, with the results presented in **Supplementary Figure 13**.

In the FAERS database, the term drug ineffective is the most frequently reported among all PTs (**Figure 7A**). In the JADER database, the term toxicity to various agents is the most frequently reported among all PTs (**Figure 7B**). PTs were classified the PTs data by SOC categories and selected Nervous system disorders and Blood and lymphatic system disorders as the SOC for analysis. In the FAERS database, migraine and bicytopenia showed the strongest correlation (**Figures 7C, D**).

**Figure 7:**
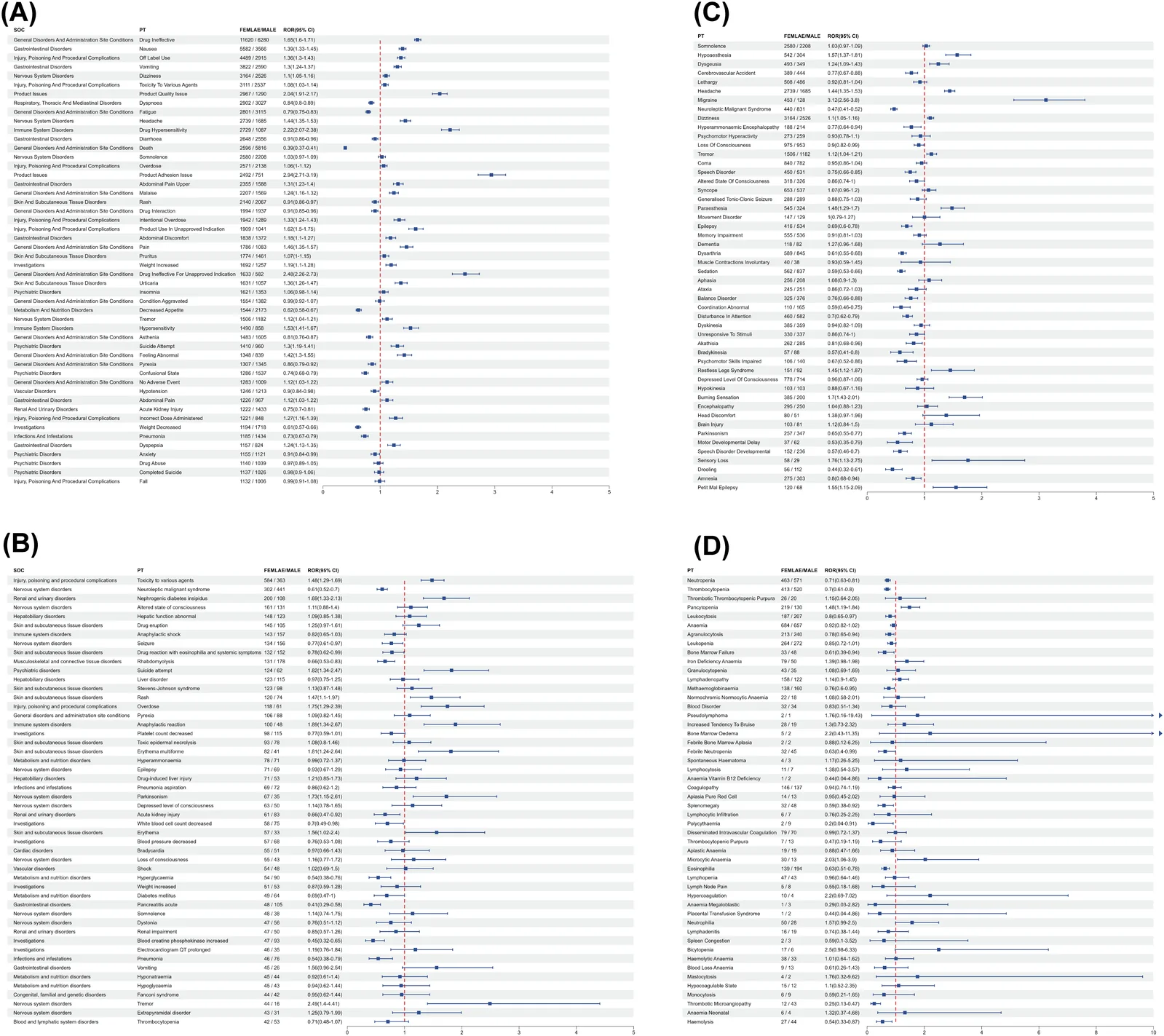
Male and female subgroup analysis at the SOCs and PTs level. **(A)**. Male and female subgroup analysis of all SOCs in FAERS database **(B)**. Male and female subgroup analysis of all SOCs in JADER database **(C)**. Male and female subgroup analysis of nervous system disorders SOCs in FAERS database **(D)**. Male and female subgroup analysis of blood and lymphatic system disorders SOCs in FAERS database.

### 3.3 GEO database analysis

#### 3.3.1 Transcriptional changes in neurons induced by GSK-3β inhibitors

To further investigate the specific mechanisms underlying the adverse effects of GSK-3β inhibitors, transcriptomic data were retrieved transcriptomic data of human neuronal cell lines treated with GSK-3β inhibitors from the GEO database (GSE290452) and performed further analysis (50). Differential expression analysis (p < 0.05) identified a total of 60 significantly differentially expressed genes, of which 42 were downregulated and 18 were upregulated (**Figures 8A, B**).

**Figure 8:**
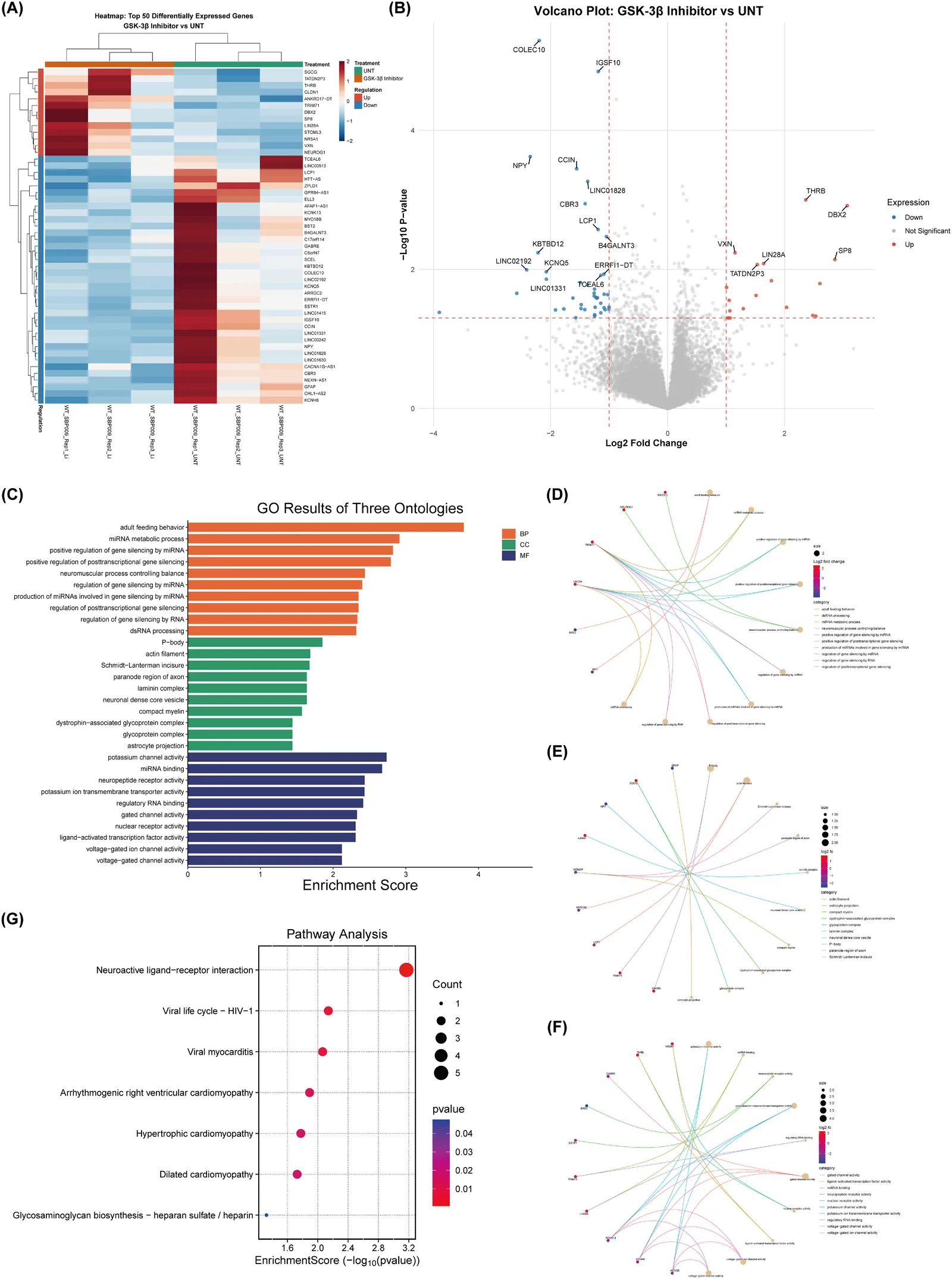
Transcriptional Changes Induced by GSK-3β Inhibitor Treatment in Normal Human Neuronal Cells. **(A)** Heatmap of differentially expressed genes. A hierarchical clustering heatmap shows the expression profiles of 60 significantly dysregulated genes (p < 0.05) in all treatment groups (Li) and the control group (UNT), with Z-score normalization. Gene names are labeled on the right. **(B)** Volcano plot of differentially expressed genes. Each point represents a gene. Significantly upregulated (log_2_FC > 1, p < 0.05) and downregulated (log_2_FC < -1, p < 0.05) genes are highlighted in red and blue, respectively. Genes that did not meet the significance threshold are shown in gray. Key genes are labeled. **(C)** Gene Ontology (GO) enrichment analysis of differentially expressed genes. The bar chart summarizes the most significantly enriched GO terms (p < 0.05) in three categories: Biological Process (BP), Cellular Component (CC), and Molecular Function (MF), ordered by enrichment score (-log_10_(p-value)). The number of genes enriched in each term (Count) is indicated. **(D)** Significantly enriched GO-BP terms and their associated gene network. The network graph displays the significant biological process terms (p < 0.05) and their directly related differentially expressed genes. Node size represents the enrichment significance of the terms. **(E)** Significantly enriched GO-CC terms and their associated gene network. The network graph displays the significant cellular component terms (p < 0.05) and their directly related differentially expressed genes. Node size represents the enrichment significance of the terms. **(F)** Significantly enriched GO-MF terms and their associated gene network. The network graph displays the significant molecular function terms (p < 0.05) and their directly related differentially expressed genes. Node size represents the enrichment significance of the terms. **(G)** KEGG pathway enrichment analysis of differentially expressed genes. The dot plot presents the results of KEGG pathway enrichment analysis. The size of the dots represents the number of genes enriched in the pathway (Count), and the color indicates statistical significance (-log_10_(p-value)). The X-axis shows the enrichment ratio.

#### 3.3.2 GO analysis

GO enrichment analysis encompassed three categories: Biological Process (BP), Cellular Component (CC), and Molecular Function (MF) (**Figure 8C**). The GO-BP analysis identified a large number of significantly enriched terms related to neural function, development, and regulation (**Figure 8D**). Among them, the most significantly enriched term was “adult feeding behavior”(GO:0008343, p = 1.60×10-4), involving Neuropeptide Y (NPY) and Bombesin-like receptor 3 (BRS3). Several terms associated with miRNA metabolism and gene silencing regulation were also significantly enriched, such as “miRNA metabolic process” (GO:0010586, p = 1.22×10-3) and “regulation of gene silencing by miRNA” (GO:0060964, p = 3.99×10-3), mainly driven by LIN28A and TRIM71. In addition, a number of processes related to neural development and function, including “neuromuscular process controlling balance”(GO:0050885, p = 3.68×10-3), “gliogenesis” (GO:0042063, p = 1.99×10-2), “regulation of neurogenesis” (GO:0050767, p = 3.24×10-2), “G protein-coupled receptor signaling pathway, coupled to cyclic nucleotide second messenger” (GO:0007187, p = 7.35×10-3), and “potassium ion transmembrane transport” (GO:0071805, p = 8.48×10-3), all showed significant enrichment.

GO-CC analysis showed that the differentially expressed genes were significantly enriched in neuronal and subcellular structures (**Figure 8E**). The results indicated that the differentially expressed genes were significantly enriched in “P-body” (GO:0000932, p = 1.41×10^-2^), including LIN28A and TRIM71. Cytoskeleton-related “actin filament” (GO:0005884, p = 2.07×10^-2^) as well as specialized neuronal structures such as “Schmidt-Lanterman incisure” (GO:0043220, p = 2.12×10^-2^) and “compact myelin” (GO:0043218, p = 2.69×10^-2^) were also significantly enriched. In addition, “laminin complex” (GO:0043256, p = 2.31×10^-2^) and “gamma-aminobutyric acid (GABA)-A receptor complex” (GO:1902711, p = 3.63×10^-2^) were identified.

GO-MF analysis showed that the functions of differentially expressed genes were strongly enriched in ion channel activity and molecular binding-related terms (**Figure 8F**). The most significantly enriched functions were “potassium channel activity” (GO:0005267, p = 1.84×10^-3^) and “miRNA binding” (GO:0035198, p = 2.13×10^-3^), corresponding to the KCNQ5, KCNH6, KCNK13 gene clusters and LIN28A and TRIM71, respectively. Other key enriched terms included “neuropeptide receptor activity” (GO:0008188, p = 3.70×10^-3^), “potassium ion transmembrane transporter activity” (GO:0015079, p = 3.71×10^-3^), “nuclear receptor activity” (GO:0004879, p = 4.91×10^-3^), and “voltage-gated ion channel activity” (GO:0005244, p = 7.63×10^-3^). Terms related to GABAergic signaling, including “GABA-A receptor activity” (GO:0004890, p = 3.76×10^-2^) and “heparan sulfate sulfotransferase activity” (GO:0034483, p = 2.98×10^-2^), were also detected.

#### 3.3.3 KEGG pathway enrichment analysis

To further elucidate the functional associations of the differentially expressed genes, KEGG pathway enrichment analysis was performed pathway enrichment analysis (**Figure 8G**). The results showed that these genes were significantly enriched in the “Neuroactive ligand-receptor interaction” pathway (hsa04080, p = 6.78×10-4), involving five genes: NPY, thyroid hormone receptor beta (THRB), somatostatin receptors 1(SSTR1), GABA receptor subunit epsilon (GABRE), and BRS3. In addition, enrichment trends were also observed in the pathways “Viral life cycle - HIV-1” (hsa03250, p = 7.25×10-3), “Viral myocarditis” (hsa05416, p = 8.62×10-3), “Arrhythmogenic right ventricular cardiomyopathy” (hsa05412, p = 1.28×10-2), and “Glycosaminoglycan biosynthesis - heparan sulfate/heparin” (hsa00534, p = 4.75×10-2).

### 3.4 Experimental validation of GSK-3β inhibitor effects

To complement the pharmacovigilance and transcriptomic analyses, SH-SY5Y cells were exposed to 9-ING-41, a direct ATP-competitive GSK-3β inhibitor (31). Cell viability, gene expression, and oxidative stress were assessed to examine biological plausibility for selected neurological and inflammatory hypotheses. These *in-vitro* results are not a clinical validation of pharmacovigilance signals.

#### 3.4.1 Cell viability

SH-SY5Y cells were exposed to increasing concentrations of 9-ING-41 (0.25–10 μM) for 24 h. Cell viability showed a concentration-associated decrease, with mean viabilities of 99.8%, 95.0%, 89.8%, 82.6%, 65.9%, and 46.9% at 0.25, 0.5, 1, 2, 5, and 10 μM, respectively. Because 1 μM 9-ING-41 maintained approximately 90% viability, this condition was selected for subsequent qRT-PCR and ROS analyses (**Figure 9A**).

**Figure 9:**
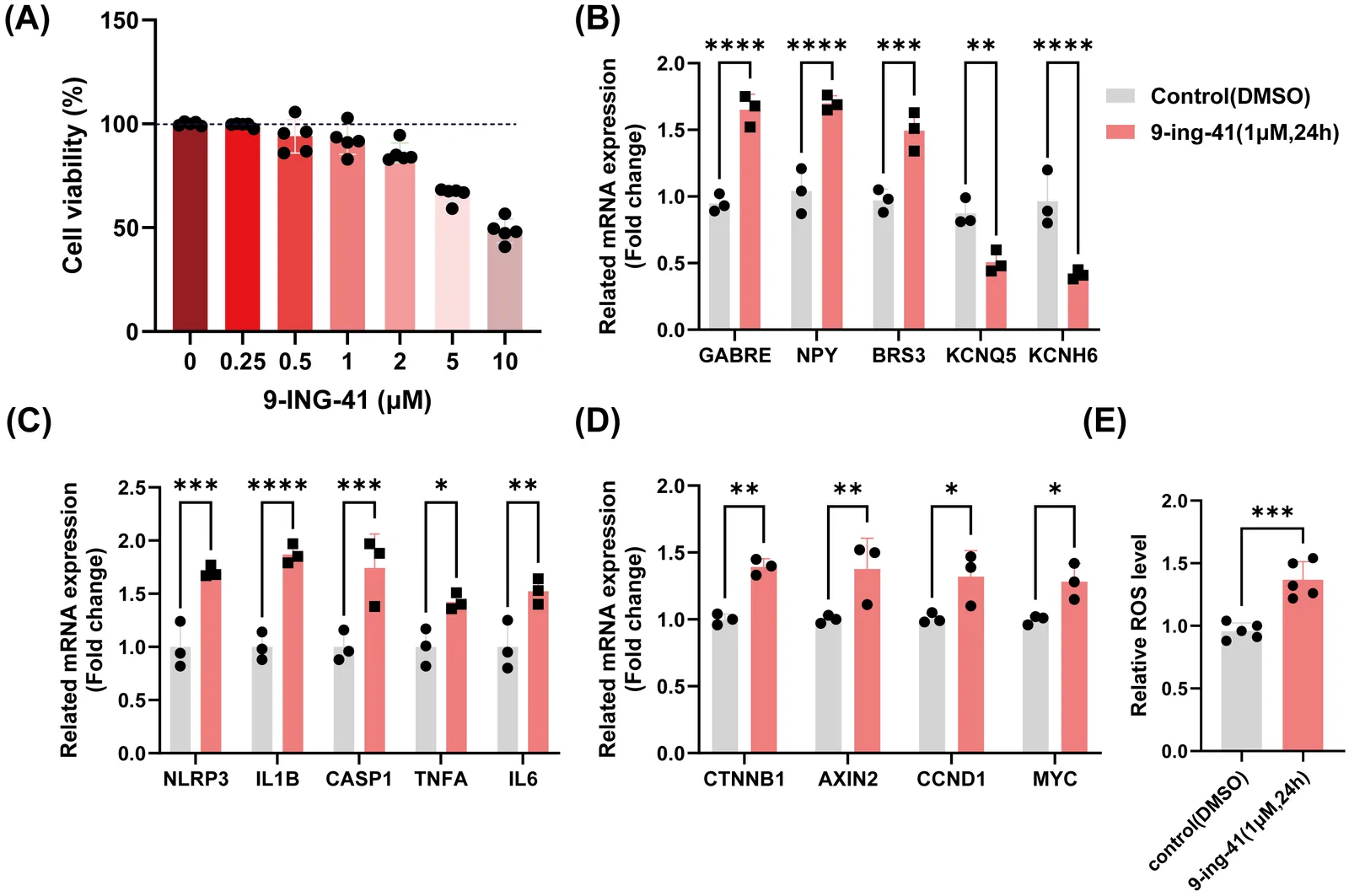
Experimental validation of transcriptomic alterations induced by GSK3β inhibition in SH-SY5Y cells. **(A)** Cell viability of SH-SY5Y cells treated with increasing concentrations of the GSK3β inhibitor 9-ING-41 (0.25–10 μM) for 24 h. Cell viability was determined using the Cell Counting Kit-8 (CCK-8) assay and expressed as a percentage relative to the untreated control group. Data are presented as mean ± SEM (n = 5). Statistical significance was determined using one-way ANOVA followed by Dunnett’s multiple comparisons test. **(B)** Relative mRNA expression levels of neuronal function-associated genes (GABRE, NPY, BRS3, KCNQ5, and KCNH6) in SH-SY5Y cells following treatment with 9-ING-41 (1 μM) for 24 h. Gene expression levels were normalized to GAPDH and calculated using the 2^-ΔΔCt method. Data are presented as mean ± SEM from three independent experiments. Statistical significance was analyzed using ordinary two-way ANOVA followed by Šídák’s multiple-comparisons test. **(C)** Relative mRNA expression levels of inflammation-associated genes (NLRP3, IL1B, CASP1, TNFA, and IL6) in SH-SY5Y cells following treatment with 9-ING-41 (1 μM) for 24 h. Gene expression levels were normalized to GAPDH and calculated using the 2^-ΔΔCt method. Data are presented as mean ± SEM from three independent experiments. Statistical significance was analyzed using ordinary two-way ANOVA followed by Šídák’s multiple-comparisons test. **(D)** Relative mRNA expression levels of GSK3β/Wnt pathway-associated genes (CTNNB1, AXIN2, CCND1, and MYC) in SH-SY5Y cells following treatment with 9-ING-41 (1 μM) for 24 h. Gene expression levels were normalized to GAPDH and calculated using the 2^-ΔΔCt method. Data are presented as mean ± SEM from three independent experiments. Statistical significance was analyzed using ordinary two-way ANOVA followed by Šídák’s multiple-comparisons test. **(E)** Intracellular reactive oxygen species (ROS) levels in SH-SY5Y cells following treatment with 9-ING-41 (1 μM) for 24 h. ROS production was assessed using the DCFH-DA fluorescent probe assay and expressed as relative fluorescence intensity normalized to the control group. Data are presented as mean ± SEM from five measurements per group. Statistical significance was analyzed using an unpaired two-tailed Student’s t-test. For all panels, statistical significance is indicated as follows: ns, not significant; *P < 0.05; **P < 0.01; ***P < 0.001;****P <0.0001.

#### 3.4.2 Neuronal function-associated genes

qRT-PCR analysis showed significant alterations in all assessed neuronal function-associated transcripts after treatment with 1 μM 9-ING-41 for 24 h. Compared with the DMSO control, GABRE, NPY, and BRS3 expression increased by 1.74-fold, 1.70-fold, and 1.54-fold, respectively (Šídák-adjusted P < 0.0001, P < 0.0001, and P = 0.0002). In contrast, KCNQ5 and KCNH6 expression decreased to 59.5% and 49.8% of control levels, respectively (adjusted P = 0.0106 and P = 0.0005). These results indicate modest changes in neuronal signaling- and ion channel-related gene expression in this cell-based model (**Figure 9B**).

#### 3.4.3 Inflammation-associated genes

Expression of inflammation-associated genes was also significantly increased following 9-ING-41 exposure. Relative to the DMSO control, NLRP3, IL1B, CASP1, TNFA, and IL6 increased by 1.62-fold, 1.71-fold, 1.72-fold, 1.38-fold, and 1.43-fold, respectively (Šídák-adjusted P = 0.0010, P = 0.0001, P = 0.0004, P = 0.0368, and P = 0.0059). These findings are compatible with low-level inflammatory transcriptional responses in SH-SY5Y cells and require confirmation in relevant neural and immune cell models (**Figure 9C**).

#### 3.4.4 GSK-3β/Wnt pathway-associated genes

In the GSK-3β/Wnt pathway panel, all four assessed transcripts were significantly increased after 9-ING-41 treatment. CTNNB1, AXIN2, CCND1, and MYC increased by 1.32-fold, 1.41-fold, 1.33-fold, and 1.44-fold, respectively (Šídák-adjusted P = 0.0291, P = 0.0037, P = 0.0133, and P = 0.0049). This expression pattern is consistent with transcriptional modulation of selected GSK-3β/Wnt pathway-associated genes; it does not, by itself, establish functional pathway activation (**Figure 9D**).

#### 3.4.5 Intracellular ROS

Intracellular ROS levels were increased following treatment with 1 μM 9-ING-41 for 24 h. DCFH-DA fluorescence increased by approximately 1.46-fold relative to the control group (unpaired two-tailed t-test, P = 0.0007), indicating oxidative-stress signaling in this *in-vitro* model (**Figure 9E**).

## 4 Discussion

### 4.1 A multi-dimensional framework for evaluating GSK-3β inhibitors

This study combines bibliometric analysis with three independent pharmacovigilance datasets (FAERS, JADER, and CVARD) and a cell-based experiment. The framework juxtaposes research activity with exploratory reporting patterns and biological plausibility. It is intended to generate and prioritize safety hypotheses for subsequent epidemiological, pharmacological, and clinical evaluation, rather than to determine causal risk.

### 4.2 Rising scholarly attention mirrors—but lags behind—real-world safety concerns

Publication output on GSK-3β inhibitors have grown steadily since 1999, accelerating in high-impact titles including Cancer Research, PLoS ONE and Clinical Cancer Research. Concomitantly, pharmacovigilance reports for agents with GSK-3β activity have surged, peaking in 2020—two decades after the initial publication uptick. This time-lag underscores a familiar pattern: mechanistic enthusiasm often precedes systematic safety interrogation.

Keyword enrichment analysis cement this narrative. Classical mechanistic terms—”GSK-3β”, “apoptosis”, “protein kinase”—occupy the motor-themes quadrant, reflecting high maturity and centrality. Newer clinical phrases—”anticancer agents”, “radiotherapy”—have only recently emerged, signaling a pivot toward translational application. Yet descriptors of toxicity (“neurotoxicity”, “hematotoxicity”) are conspicuously absent from keyword clusters despite being among the most frequent signals in FAERS, JADER and CVARD. The field therefore risks repeating a familiar pitfall: efficacy first, safety later.

### 4.3 Neurological toxicity: paradoxes and clinical implications

GSK-3β inhibition has been investigated for neuroprotective effects in models of Alzheimer’s and Parkinson’s disease (51, 52). In the present datasets, nervous-system disorders generated recurrent disproportionality signals, with dizziness, somnolence, and headache among the more frequently reported PTs in FAERS and CVARD. Olanzapine showed a prominent sedation signal, but its established receptor pharmacology and potential co-medications preclude attribution of this finding to GSK-3β inhibition alone. These data support careful neurological assessment in future prospective studies; they do not establish a class-wide causal effect.

This seemingly paradoxical phenomenon stems from the highly context-dependent dual role of GSK-3β in the regulation of central nervous system functions. Fundamental research has unveiled a complex “bidirectional modulation” effect of GSK-3β inhibitors on pain perception (53). In models of bone cancer pain, inhibitors like kenpaullone can enhance K^(+)^-Cl (–) co-transporter 2 (KCC2) expression by promoting the nuclear translocation of δ-catenin, thereby restoring GABAergic synaptic transmission at the spinal cord level (54). Alternatively, 4-benzyl-2-methyl-1,2,4-thiadiazolidine-3,5-dione (TDZD-8) can alleviate neuroinflammation by inhibiting dynamin-related protein 1 (DRP1)-mediated mitochondrial hyperfusion and subsequent activation of the Nucleotide oligomerization domain (NOD)-like receptor protein 3 (NLRP3) inflammasome, ultimately exerting analgesic effects (55–57). However, in models of peripheral nerve injury, inhibition of GSK-3β (such as with SB216763) can directly induce persistent hyperalgesia (58). This discrepancy may be attributed to the differing pathological bases of various pain models (such as inflammation-driven versus neuropathically-driven), the specific functions of GSK-3β in different neuronal cell types and microcircuits, as well as the spatiotemporal dynamics of therapeutic interventions.

Transcriptomic analysis of lithium-treated neuronal cultures identified changes in neurofunctional pathways (59, 60). In the SH-SY5Y experiment, 1 μM 9-ING-41 for 24 h modestly altered neuronal function-associated genes, mildly increased inflammatory-gene expression, increased selected GSK-3β/Wnt-related transcripts and intracellular ROS while maintaining approximately 90% viability. These observations provide preliminary biological plausibility for the hypotheses generated by the spontaneous-reporting analyses; they are not evidence that the reported neurological events were caused by GSK-3β inhibition in patients.

The enrichment of glial-related genes (for example, glial fibrillary acidic protein [GFAP] and LIN28A) raises the possibility that non-neuronal cells contribute to the synaptic microenvironment (61, 62). This inference should be tested in cell-type-resolved models. It cannot establish a universal regulatory role or a class effect from the current transcriptomic and *in-vitro* datasets.

The findings suggest a rationale for testing cell-type- or pathway-selective GSK-3β modulation and for examining neurological outcomes prospectively. They do not explain the sedative effects of olanzapine or establish that GSK-3β activity is the mechanism of those effects. Future work should separate target-mediated effects from each agent’s established pharmacology, treatment indication, and co-medications.

### 4.4 Hematologic and coagulation liabilities

Anemia, neutropenia, and thrombocytopenia appeared among recurrent blood/lymphatic-system PTs across the three databases. These observations may reflect the underlying disease, anticancer therapy, co-medications, or differential reporting as well as any drug-related effects; they should therefore be treated as safety hypotheses rather than a class effect. Lidocaine-associated methemoglobinemia produced a strong disproportionality signal, but this finding should not be interpreted as evidence of a GSK-3β-mediated redox mechanism. Prospective studies should include clinically appropriate hematological and coagulation surveillance when evaluating investigational GSK-3β-directed regimens.

### 4.5 From enthusiasm to stewardship

Collectively, the results support a balanced development strategy: antitumor hypotheses should be evaluated alongside prospective neurological and hematological safety assessments. Coordinated pharmacovigilance, mechanistic experiments, and early-phase trial safety endpoints can help prioritize signals for formal evaluation, while avoiding causal interpretation of spontaneous-reporting associations.

### 4.6 Clinical implications

For development or clinical investigation of GSK-3β-directed regimens, baseline neurological history, concomitant sedating medicines, and longitudinal assessment of dizziness, somnolence, and altered consciousness should be considered. Blood counts and, when clinically indicated, coagulation-related monitoring may help identify potentially vulnerable patients. These are risk-management considerations, not recommendations based on proven causal effects. In a recent phase II pancreatic-cancer trial, elraglusib was evaluated with gemcitabine and nab-paclitaxel; reported neutropenia and anemia in combination therapy reinforce the importance of prospective safety monitoring while not isolating the effect of elraglusib from chemotherapy (63).

### 4.7 Limitations

This study has several limitations. First, FAERS, JADER, and CVARD are spontaneous-reporting systems subject to under-reporting, selective reporting, duplicate or incomplete reports, and missing demographic and exposure information (49). Second, disproportionality methods do not estimate incidence and cannot establish causality because confounding by indication, disease severity, concomitant medication, and differential reporting may remain even after adjustment (22). Third, the study-drug panel includes agents with heterogeneous and, for some agents, lower-certainty GSK-3β activity; cross-drug findings must not be interpreted as effects of a pharmacologically uniform class. Fourth, TTO analysis was limited to FAERS because compatible start- and onset-date fields were unavailable in JADER and CVARD. Finally, the transcriptomic and SH-SY5Y experiments used a single exposure condition and cell context and therefore provide preliminary biological plausibility rather than clinical validation.

## 5 Conclusion

This integrated analysis maps the GSK-3β inhibitor research landscape and identifies exploratory neurological and hematological reporting signals across three spontaneous-reporting databases. Cross-database consistency can prioritize hypotheses, whereas database-specific patterns and the heterogeneous pharmacology of the study drugs require caution. The 9-ING-41 cell model provides preliminary support for neurofunctional, inflammatory, and oxidative-stress hypotheses, but prospective, controlled studies are required to estimate incidence and establish causality.

## Data Availability

The raw data supporting the conclusions of this article will be made available by the authors, without undue reservation.

## Glossary

GBD: global burden of disease
CAR-T: chimeric antigen receptor T
GSK-3β: Glycogen synthase kinase 3 Beta
FAERS: U.S. Food and Drug Administration Adverse Event Reporting System
JADER: Japanese Adverse Drug Event Report
CVARD: Canadian Vigilance Adverse Reaction Database
ADRs: adverse drug reactions
SOC: System Organ Class
PT: Preferred Term
HLGT: High Level Group Term
HLT: High Level Term
TTO: time-to-onset
WoSCC: Core Collection of Web of Science
JCR: Journal Citation Reports
ROR: Reporting Odds Ratio
PRR: Proportional Reporting Ratio
BCPNN: Bayesian Confidence Propagation Neural Network
EBGM: Empirical Bayes Geometric Mean
AEs: Adverse events
IMEs: important medical events
DMEs: designated medical events
CI: confidence interval
GEO: Gene Expression Omnibus
DEGs: differentially expressed genes
GO: Gene Ontology
KEGG: Kyoto Encyclopedia of Genes and Genomes
MCP: multi-country publication
SCP: single-country publications
BP: Biological Process
CC: Cellular Component
MF: Molecular Function
NPY: Neuropeptide Y
BRS3: Bombesin-like receptor 3
THRB: thyroid hormone receptor beta
SSTR1: somatostatin receptors 1
GABA: gamma-aminobutyric acid
GABRE: GABA receptor subunit epsilon
KCC2: K^(+)^-Cl^(-)^ co-transporter 2
TDZD-8: 4-benzyl-2-methyl-1,2,4-thiadiazolidine-3,5-dione
DRP1: dynamin-related protein 1
NOD: Nucleotide oligomerization domain
NLRP3: NOD-like receptor protein 3
GFAP: glial fibrillary acidic protein
