# Supplementary material for: Neurological and hematological safety profiles of GSK-3β inhibitors: insights from multi-database pharmacovigilance and experimental validation
Source: Front Immunol. 2026 Sep 17;17:1915614. doi: 10.3389/fimmu.2026.1915614 (PMC13627430; doi:10.3389/fimmu.2026.1915614)
Supplement: Supplementary file 1 [file DataSheet1.zip › Supplementary Tables S3-S13.DOCX]

Table S3. Four grid table

|  | GSK 3βinhibitor-related ADEs | Non-GSK 3βinhibitor-related ADEs | Total |
| --- | --- | --- | --- |
| GSK 3βinhibitor | a | b | a+b |
| Non GSK 3βinhibitor | c | d | c+d |
| Total | a+c | b+d | N=a+b+c+d |

Table S4. The specific equations for four methodologies

| Methodologies | Equation | Threshold |
| --- | --- | --- |
| ROR | ROR= $\frac{a/c}{b/d}$ | a$\geq$3 and 95% CI (lower limit) > 1 |
|  | *SE*(*ln*ROR)=$\sqrt{\frac{1}{a}+\frac{1}{b}+\frac{1}{c}+\frac{1}{d}}$ |  |
|  | 95%*CI*=$e^{ln(ROR)\pm1.96\sqrt{\frac{1}{a}+\frac{1}{b}+\frac{1}{c}+\frac{1}{d}}}$ |  |
| PRR | PRR=$\frac{a/(a+b)}{c/(c+d)}$ | a$\geq$3 and 95% CI (lower limit) > 1 |
|  | *SE*(*ln*PRR)=$\sqrt{\frac{1}{a}-\frac{1}{a+b}+\frac{1}{c}-\frac{1}{c+d}}$ |  |
|  | 95%*CI*=$e^{ln(PRR)\pm1.96\sqrt{\frac{1}{a}-\frac{1}{a+b}+\frac{1}{c}-\frac{1}{c+d}}}$ |  |
| BCPNN | IC=${log}_{2}\frac{p(x,y)}{p\left( x \right)p(y)}$=${log}_{2}\frac{a(a+b+c+d)}{(a+b)(a+c)}$ | IC025 > 0 |
|  | IC-2SD=E(IC)-2$\sqrt{V(IC)}$ |  |
| EBGM | EBGM=$\frac{a/(a+b+c+d)}{(a+c)/(a+b)}$ | EBGM05 > 2 |
|  | 95%IC=$e^{ln(EBGM)\pm1.96\sqrt{\frac{1}{a}+\frac{1}{b}+\frac{1}{c}+\frac{1}{d}}}$ |  |

Table S5. Criteria and relevant scores to prioritize AEs emerged from disproportionality analysis

| Criterium | 2 points | 1 point | 0 point |
| --- | --- | --- | --- |
| Reporting rate (cases/non-cases) | > 10% | 1–10% | 0–1% |
| Signal stability (consistency across disproportionality analyses) | 3 of 3 | 2 of 3 | 1 of 3 |
| Reported case fatality rate (proportion of reports with death as outcome) | > 50% | 25–50% | < 25% |
| Clinical relevance (serious likely drug-attributable AEs) | designated medical event(DME) | important medical event(IME) | None |

Table S6.Nervous system disorders AERs ranked as high clinical priorities and relevant scores in FAERS database (TOP 30)

| **PT** | **Score of reporting rate** | **Score of signal stability** | **Score of reported case fatality rate** | **Score of clinical relevance** | **Total score** | **Clinical** priority |
| --- | --- | --- | --- | --- | --- | --- |
| Brain Oedema | 0 | 2 | 2 | 1 | 5 | moderate priority |
| Cytotoxic Oedema | 0 | 2 | 2 | 1 | 5 | moderate priority |
| Hyponatraemic Encephalopathy | 0 | 2 | 2 | 1 | 5 | moderate priority |
| Intraventricular Haemorrhage | 0 | 2 | 1 | 1 | 4 | moderate priority |
| Hypoxic-Ischaemic Encephalopathy | 0 | 2 | 1 | 1 | 4 | moderate priority |
| Coma Hepatic | 0 | 2 | 1 | 1 | 4 | moderate priority |
| Neurodegenerative Disorder | 0 | 2 | 1 | 1 | 4 | moderate priority |
| Periventricular Leukomalacia | 0 | 2 | 1 | 1 | 4 | moderate priority |
| Intraventricular Haemorrhage Neonatal | 0 | 2 | 1 | 1 | 4 | moderate priority |
| Progressive Supranuclear Palsy | 0 | 2 | 1 | 1 | 4 | moderate priority |
| Vascular Encephalopathy | 0 | 2 | 1 | 1 | 4 | moderate priority |
| Cytotoxic Lesions Of Corpus Callosum | 0 | 2 | 1 | 1 | 4 | moderate priority |
| Basilar Artery Occlusion | 0 | 2 | 1 | 1 | 4 | moderate priority |
| Encephalopathy Neonatal | 0 | 2 | 1 | 1 | 4 | moderate priority |
| Ischaemic Neuropathy | 0 | 2 | 1 | 1 | 4 | moderate priority |
| Hypokinesia Neonatal | 0 | 2 | 1 | 1 | 4 | moderate priority |
| Coma | 0 | 2 | 0 | 1 | 3 | moderate priority |
| Depressed Level Of Consciousness | 0 | 2 | 0 | 1 | 3 | moderate priority |
| Neuroleptic Malignant Syndrome | 0 | 2 | 0 | 1 | 3 | moderate priority |
| Epilepsy | 0 | 2 | 0 | 1 | 3 | moderate priority |
| Post-Injection Delirium Sedation Syndrome | 0 | 2 | 0 | 1 | 3 | moderate priority |
| Unresponsive To Stimuli | 0 | 2 | 0 | 1 | 3 | moderate priority |
| Altered State Of Consciousness | 0 | 2 | 0 | 1 | 3 | moderate priority |
| Generalised Tonic-Clonic Seizure | 0 | 2 | 0 | 1 | 3 | moderate priority |
| Encephalopathy | 0 | 2 | 0 | 1 | 3 | moderate priority |
| Dystonia | 0 | 2 | 0 | 1 | 3 | moderate priority |
| Hyperammonaemic Encephalopathy | 0 | 2 | 0 | 1 | 3 | moderate priority |
| Serotonin Syndrome | 0 | 2 | 0 | 1 | 3 | moderate priority |
| Neurotoxicity | 0 | 2 | 0 | 1 | 3 | moderate priority |
| Status Epilepticus | 0 | 2 | 0 | 1 | 3 | moderate priority |

Table S7.Blood and lymphatic system disorders AERs ranked as high clinical priorities and relevant scores in FAERS database

| **PT** | **Score of reporting rate** | **Score of signal stability** | **Score of reported case fatality rate** | **Score of clinical relevance** | **Total score** | **Clinical priority** |
| --- | --- | --- | --- | --- | --- | --- |
| Agranulocytosis | 0 | 2 | 0 | 2 | 4 | moderate priority |
| Hyperleukocytosis | 0 | 2 | 1 | 1 | 4 | moderate priority |
| Spleen Congestion | 0 | 2 | 2 | 0 | 4 | moderate priority |
| Mast Cell Activation Syndrome | 0 | 2 | 0 | 1 | 3 | moderate priority |
| Monocytosis | 0 | 2 | 1 | 0 | 3 | moderate priority |
| Evans Syndrome | 0 | 2 | 0 | 1 | 3 | moderate priority |
| Hypoplastic Anaemia | 0 | 2 | 0 | 1 | 3 | moderate priority |
| Coagulation Disorder Neonatal | 0 | 2 | 0 | 1 | 3 | moderate priority |
| Granulocytes Maturation Arrest | 0 | 2 | 0 | 1 | 3 | moderate priority |
| Leukocytosis | 0 | 2 | 0 | 0 | 2 | weak priority |
| Eosinophilia | 0 | 2 | 0 | 0 | 2 | weak priority |
| Methaemoglobinaemia | 0 | 2 | 0 | 0 | 2 | weak priority |
| Coagulopathy | 0 | 1 | 1 | 0 | 2 | weak priority |
| Microcytic Anaemia | 0 | 2 | 0 | 0 | 2 | weak priority |
| Normocytic Anaemia | 0 | 2 | 0 | 0 | 2 | weak priority |
| Hypocoagulable State | 0 | 2 | 0 | 0 | 2 | weak priority |
| Thrombocytopenic Purpura | 0 | 1 | 0 | 1 | 2 | weak priority |
| Hypofibrinogenaemia | 0 | 2 | 0 | 0 | 2 | weak priority |
| Abnormal Clotting Factor | 0 | 2 | 0 | 0 | 2 | weak priority |
| Normochromic Anaemia | 0 | 2 | 0 | 0 | 2 | weak priority |
| Splenic Lesion | 0 | 2 | 0 | 0 | 2 | weak priority |
| Platelet Aggregation Inhibition | 0 | 2 | 0 | 0 | 2 | weak priority |
| Benign Ethnic Neutropenia | 0 | 2 | 0 | 0 | 2 | weak priority |
| Spherocytic Anaemia | 0 | 2 | 0 | 0 | 2 | weak priority |
| Haemorrhagic Disease Of Newborn | 0 | 0 | 1 | 1 | 2 | weak priority |
| Iron Deficiency Anaemia | 0 | 1 | 0 | 0 | 1 | weak priority |
| Neutrophilia | 0 | 1 | 0 | 0 | 1 | weak priority |
| Hyperchromasia | 0 | 0 | 0 | 0 | 0 | weak priority |

Table S8.Nervous system disorders AERs ranked as high clinical priorities and relevant scores in JADER database (TOP 30)

| **PT** | **Score of reporting rate** | **Score of signal stability** | **Score of reported case fatality rate** | **Score of clinical relevance** | **Total score** | **Clinical priority** |
| --- | --- | --- | --- | --- | --- | --- |
| Postresuscitation Encephalopathy | 0 | 2 | 2 | 1 | 5 | moderate priority |
| Neuroleptic Malignant Syndrome | 1 | 2 | 0 | 1 | 4 | moderate priority |
| Altered State Of Consciousness | 1 | 2 | 0 | 1 | 4 | moderate priority |
| Seizure | 1 | 2 | 0 | 1 | 4 | moderate priority |
| Diabetic Coma | 0 | 2 | 1 | 1 | 4 | moderate priority |
| Coma Hepatic | 0 | 2 | 1 | 1 | 4 | moderate priority |
| Epilepsy | 0 | 2 | 0 | 1 | 3 | moderate priority |
| Dystonia | 0 | 2 | 0 | 1 | 3 | moderate priority |
| Tardive Dyskinesia | 0 | 2 | 0 | 1 | 3 | moderate priority |
| Generalised Tonic-Clonic Seizure | 0 | 2 | 0 | 1 | 3 | moderate priority |
| Serotonin Syndrome | 0 | 2 | 0 | 1 | 3 | moderate priority |
| Status Epilepticus | 0 | 2 | 0 | 1 | 3 | moderate priority |
| Intraventricular Haemorrhage | 0 | 2 | 0 | 1 | 3 | moderate priority |
| Coma | 0 | 2 | 0 | 1 | 3 | moderate priority |
| Hyperammonaemic Encephalopathy | 0 | 2 | 0 | 1 | 3 | moderate priority |
| Hypoxic-Ischaemic Encephalopathy | 0 | 2 | 0 | 1 | 3 | moderate priority |
| Tonic Convulsion | 0 | 2 | 0 | 1 | 3 | moderate priority |
| Cerebral Atrophy | 0 | 2 | 0 | 1 | 3 | moderate priority |
| Myoclonic Epilepsy | 0 | 2 | 0 | 1 | 3 | moderate priority |
| Akinesia | 0 | 2 | 0 | 1 | 3 | moderate priority |
| Petit Mal Epilepsy | 0 | 2 | 0 | 1 | 3 | moderate priority |
| Cauda Equina Syndrome | 0 | 2 | 0 | 1 | 3 | moderate priority |
| Dementia With Lewy Bodies | 0 | 2 | 0 | 1 | 3 | moderate priority |
| Seizure Cluster | 0 | 2 | 0 | 1 | 3 | moderate priority |
| Partial Seizures | 0 | 2 | 0 | 1 | 3 | moderate priority |
| Posthaemorrhagic Hydrocephalus | 0 | 2 | 0 | 1 | 3 | moderate priority |
| Diabetic Hyperosmolar Coma | 0 | 2 | 0 | 1 | 3 | moderate priority |
| Diplegia | 0 | 2 | 0 | 1 | 3 | moderate priority |
| Cerebellar Atrophy | 0 | 2 | 0 | 1 | 3 | moderate priority |
| Intellectual Disability | 0 | 2 | 0 | 1 | 3 | moderate priority |

Table S9. Blood and lymphatic system disorders AERs ranked as high clinical priorities and relevant scores in JADER database

| **PT** | **Score of reporting rate** | **Score of signal stability** | **Score of reported case fatality rate** | **Score of clinical relevance** | **Total score** | **Clinical priority** |
| --- | --- | --- | --- | --- | --- | --- |
| Aplastic Anaemia | 0 | 1 | 0 | 2 | 3 | moderate priority |
| Aplasia Pure Red Cell | 0 | 1 | 0 | 2 | 3 | Moderate priority |
| Granulocytopenia | 0 | 0 | 0 | 2 | 2 | weak priority |
| Methaemoglobinaemia | 0 | 2 | 0 | 0 | 2 | weak priority |

Table S10.Nervous system disorders AERs ranked as high clinical priorities and relevant scores in CVARD database (TOP 30)

| **PT** | **Score of reporting rate** | **Score of signal stability** | **Score of reported case fatality rate** | **Score of clinical relevance** | **Total score** | **Clinical priority** |
| --- | --- | --- | --- | --- | --- | --- |
| Cerebral Hypoperfusion | 0 | 2 | 2 | 1 | 5 | moderate priority |
| Hypoxic-Ischaemic Encephalopathy | 0 | 2 | 1 | 1 | 4 | moderate priority |
| Apallic Syndrome | 0 | 2 | 1 | 1 | 4 | moderate priority |
| Seizure | 0 | 2 | 0 | 1 | 3 | moderate priority |
| Depressed Level Of Consciousness | 0 | 2 | 0 | 1 | 3 | moderate priority |
| Dystonia | 0 | 2 | 0 | 1 | 3 | moderate priority |
| Neuroleptic Malignant Syndrome | 0 | 2 | 0 | 1 | 3 | moderate priority |
| Coma | 0 | 2 | 0 | 1 | 3 | moderate priority |
| Generalised Tonic-Clonic Seizure | 0 | 2 | 0 | 1 | 3 | moderate priority |
| Serotonin Syndrome | 0 | 2 | 0 | 1 | 3 | moderate priority |
| Tardive Dyskinesia | 0 | 2 | 0 | 1 | 3 | moderate priority |
| Status Epilepticus | 0 | 2 | 0 | 1 | 3 | moderate priority |
| Brain Injury | 0 | 2 | 0 | 1 | 3 | moderate priority |
| Brain Oedema | 0 | 1 | 1 | 1 | 3 | moderate priority |
| Cataplexy | 0 | 2 | 0 | 1 | 3 | moderate priority |
| Partial Seizures | 0 | 2 | 0 | 1 | 3 | moderate priority |
| Diabetic Coma | 0 | 2 | 0 | 1 | 3 | moderate priority |
| Anticholinergic Syndrome | 0 | 2 | 0 | 1 | 3 | moderate priority |
| Cerebral Atrophy | 0 | 2 | 0 | 1 | 3 | moderate priority |
| Toxic Encephalopathy | 0 | 2 | 0 | 1 | 3 | moderate priority |
| Narcolepsy | 0 | 2 | 0 | 1 | 3 | moderate priority |
| Hyperammonaemic Encephalopathy | 0 | 2 | 0 | 1 | 3 | moderate priority |
| Asterixis | 0 | 2 | 0 | 1 | 3 | moderate priority |
| Intellectual Disability | 0 | 2 | 0 | 1 | 3 | moderate priority |
| Consciousness Fluctuating | 0 | 2 | 0 | 1 | 3 | moderate priority |
| Diabetic Hyperosmolar Coma | 0 | 2 | 0 | 1 | 3 | moderate priority |
| Opisthotonus | 0 | 2 | 0 | 1 | 3 | moderate priority |
| Epilepsia Partialis Continua | 0 | 2 | 0 | 1 | 3 | moderate priority |
| Encephalitis Autoimmune | 0 | 2 | 0 | 1 | 3 | moderate priority |
| Lacunar Infarction | 0 | 2 | 0 | 1 | 3 | moderate priority |

Table S11. Blood and lymphatic system disorders AERs ranked as high clinical priorities and relevant scores in CVARD database

| **PT** | **Score of reporting rate** | **Score of signal stability** | **Score of reported case fatality rate** | **Score of clinical relevance** | **Total score** | **Clinical priority** |
| --- | --- | --- | --- | --- | --- | --- |
| Agranulocytosis | 0 | 0 | 0 | 2 | 2 | weak priority |
| Methaemoglobinaemia | 0 | 2 | 0 | 0 | 2 | weak priority |
| Lymphadenopathy Mediastinal | 0 | 2 | 0 | 0 | 2 | weak priority |
| Anaemia Macrocytic | 0 | 2 | 0 | 0 | 2 | weak priority |
| Normocytic Anaemia | 0 | 2 | 0 | 0 | 2 | weak priority |
| Lymph Node Calcification | 0 | 2 | 0 | 0 | 2 | weak priority |
| Hypochromic Anaemia | 0 | 2 | 0 | 0 | 2 | weak priority |
| Spleen Atrophy | 0 | 2 | 0 | 0 | 2 | weak priority |
| Leukopenia | 0 | 0 | 0 | 1 | 1 | weak priority |
| Macrocytosis | 0 | 1 | 0 | 0 | 1 | weak priority |
| Platelet Production Decreased | 0 | 1 | 0 | 0 | 1 | weak priority |
| Leukocytosis | 0 | 0 | 0 | 0 | 0 | weak priority |
| Splenic Granuloma | 0 | 0 | 0 | 0 | 0 | weak priority |

Table S12 Time-to-onset analysis for signals of nervous system disorders SOC with strong/moderate/weak prioritization in FAERS database

| Prioritization | TTO (days) |  |  | Weibull distribution |  |  |  | Failure type |
| --- | --- | --- | --- | --- | --- | --- | --- | --- |
|  | Cases |  |  | Scale parameter |  | Shape parameter |  |  |
|  | n | Median (IQR) | Min–max | α | 95% CI | β | 95% CI |  |
| Moderate | 1395 | 31(3.5-375) | 1-15279 | 143.64 | (123.71-163.57) | 0.40 | (0.39-0.42) | Early failure |
| Weak | 5072 | 51(7-365) | 1-15857 | 170.93 | (159.45-182.41) | 0.43 | (0.43-0.44) | Early failure |

Table S13 Time-to-onset analysis for signals of blood and lymphatic system disorders SOC with strong/moderate/weak prioritization in FAERS database

| Prioritization | TTO (days) |  |  | Weibull distribution |  |  |  | Failure type |
| --- | --- | --- | --- | --- | --- | --- | --- | --- |
|  | Cases |  |  | Scale parameter |  | Shape parameter |  |  |
|  | n | Median (IQR) | Min–max | α | 95% CI | β | 95% CI |  |
| Moderate | 157 | 8(3-19) | 1-702 | 19.18 | (14.15-24.22) | 0.63 | (0.56-0.70) | Early failure |
| Weak | 254 | 13(4-61) | 1-4779 | 47.05 | (33.79-60.30) | 0.46 | (0.42-0.51) | Early failure |
