# Supplementary material for: Neurological and hematological safety profiles of GSK-3β inhibitors: insights from multi-database pharmacovigilance and experimental validation
Source: Front Immunol. 2026 Sep 17;17:1915614. doi: 10.3389/fimmu.2026.1915614 (PMC13627430; doi:10.3389/fimmu.2026.1915614)
Supplement: Supplementary file 1 [file DataSheet1.zip › Supplementary Figure Legends (S1-S13).DOCX]

Figure S1. Annual trends of publications and adverse reaction reports related to GSK-3β inhibitors. (A) illustrates the annual number of studies published on GSK-3β inhibitors, showing a significant increase over the past decade with a peak around 2020. (B) displays the cumulative publication volume, indicating a steady rise in the number of publications over the last two decades (R² = 0.7105). (C-H) depict the annual reporting of adverse reaction events associated with GSK-3β inhibitors. Notably, reports of adverse reactions have surged in recent years, particularly around 2020, mirroring the publication trend and reflecting growing concerns about the safety profile of these inhibitors. Data for February 2025 was included, though studies published after the cutoff date were not considered. These findings highlight both the sustained research interest and the increasing awareness of the drug's safety profile.

Figure S2. Author Contributions, H-index, and Citation Analysis in GSK-3β Inhibitors Research.(A) Radar chart displaying the top ten authors by publication count in the field of GSK-3β inhibitors.(B) Radar chart representing the top ten authors with the highest H-index in local articles on GSK-3β inhibitors. (C) Radar chart showing the top ten most locally cited authors in GSK-3β inhibitors research.(D) Heat map of the annual publication volume and citation count of the top 20 authors in GSK-3β inhibitors research. (E) Visualization map of the co-cited authors analysis, highlighting key contributors such as Andrei V. Ougolkov, Richard S. Jope, Abbas Shakoori, Eléonore Beurel, Bradley W. Doble, and Hoeflich KP. (F) Author publication output analysis based on Price’s Law. A total of 1873 researchers contributed to the publication of 2127 studies, with 92.31% of authors publishing only one study. The analysis also identifies core authors based on the minimum threshold of three publications.

Figure S3. Journal Collaboration, Publication Volume, Citation Trends, and Core Journals in GSK-3β Inhibitor Research.(A) Journal collaboration network showing the interdisciplinary nature of GSK-3β inhibitors research. Red nodes represent journals focused on biological and chemical sciences, while blue nodes represent medical and clinical journals. The size of a node reflects the number of articles it represents, while the width of connections indicates the strength of collaboration.(B) Top 20 journals by publication volume on GSK-3β inhibitors. Cancer Research (10 publications, 2024 Impact Factor (IF) = 16.6) leads, followed by PLoS One (10 publications, IF = 2.6) and Clinical Cancer Research (7 publications, IF = 10.2). The journals in this list are all Q1 journals, with impact factors above 2, signifying the high-quality recognition of GSK-3β inhibitors research.(C) Top 20 journals by citation count for articles on GSK-3β inhibitors. Cancer Research (603 citations, IF = 16.6) leads, followed by J Biol Chem (503 citations, IF = 3.9) and Clin Cancer Res (323 citations, IF = 10.2). The list also includes prestigious journals such as Nature, Science, and Cell, reflecting the strong theoretical foundation and recognition of GSK-3β inhibitors research.(D) Line graph showing the publication trends over time for the top 10 journals. Cancer Research, PLoS One, and Clinical Cancer Research have shown the most significant growth in the number of publications starting around 2020.(E) Core journals in GSK-3β inhibitors research according to Bradford's Law. Core journals, including "Cancer Research" and "PLOS One," dominate the publication output, aligning with the principle that a small number of journals contribute significantly to the total research in the field.

Figure S4. Keyword Analysis in GSK3β Inhibitors Across Different Periods.(A) Strategic diagram of keywords in different sub-periods (2019–2022). Keywords such as glycogen-synthase kinase-3-beta, cancer, glycogen-synthase kinase-3, apoptosis, and protein-kinase are located in the Motor Themes quadrant, indicating high relevance and maturity. GSK-3β inhibitor, anticancer agents, therapy, and radiotherapy are also core themes, while drug, metastasis, and cell-cycle arrest are positioned in the Emerging or Declining Themes quadrant, suggesting relatively lower research intensity.(B–E) Keyword density maps for consecutive years(2019–2022) . In addition to the core topics (cancer, protein kinase, glycogen-synthase kinase-3, apoptosis, expression), other prominent research themes such as immune checkpoint blockade, chemotherapy, metastasis, and biological evaluation are highlighted.

Figure S5. Relationship Between Journal Impact Factor, Citations, References, and Author Collaboration in GSK-3β Inhibitor Research.(A) Correlation between journal IF and the average number of citations per year, showing a positive relationship.(B) Correlation between journal IF and the number of references cited, indicating that higher-impact journals tend to include more references.(C) Violin plot comparing the number of authors for papers with IF > 10 versus IF ≤ 10. Papers published in higher-impact journals generally involve more authors and exhibit a broader distribution, while lower-impact papers tend to have fewer authors. The difference is statistically significant (P < 0.0001).(D) Comparison of average impact factors of articles across different time periods, demonstrating a significant increase in recent years, likely associated with larger collaborative author teams, especially after 2018.

Figure S6. Signals detection at the SOCs level. (A). The bar chart of the reported cases of ADEs at each SOC level in FAERS database (B). The ROR values of the top 8 SOCs with the largest number of reports related to GSK-3β inhibitors in the FAERS database was visualized to make a radar map (C). The bar chart of the reported cases of ADEs at each SOC level in JADER database (D). The ROR values of the top 8 SOCs with the largest number of reports related to GSK-3βinhibitors in the JADER database was visualized to make a radar map (E). The bar chart of the reported cases of ADEs at each SOC level in CVARD database (F). The ROR values of the top 8 SOCs with the largest number of reports related to GSK-3βinhibitors in the JADER database was visualized to make a radar map

Figure S7. Bar graph of PT number and proportion of target SOCs (A). shows the number of reports of nervous system disorders AEs caused by seven different drugs in FAERS databse (B). shows the proportion of nervous system disorders disorders AEs in the total AEs caused by seven different drugs in FAERS database (C). shows the number of reports of blood and lymphatic system disorders AEs caused by seven different drugs in FAERS databse (D). shows the proportion of blood and lymphatic system disorders AEs in the total AEs caused by seven different drugs in FAERS database (E). shows the number of reports of nervous system disorders AEs caused by seven different drugs in JADER databse (F). shows the proportion of nervous system disorders disorders AEs in the total AEs caused by seven different drugs in JADER database (G). shows the number of reports of blood and lymphatic system disorders AEs caused by seven different drugs in JADER databse (H). shows the proportion of blood and lymphatic system disorders AEs in the total AEs caused by seven different drugs in JADER database (I). shows the number of reports of nervous system disorders AEs caused by seven different drugs in CVARD databse (J). shows the proportion of nervous system disorders disorders AEs in the total AEs caused by seven different drugs in CVARD database (K). shows the number of reports of blood and lymphatic system disorders AEs caused by seven different drugs in CVARD databse (L). shows the proportion of blood and lymphatic system disorders AEs in the total AEs caused by seven different drugs in CVARD database

Figure S8. Venn diagram of positive results in four different algorithms for AEs caused by seven different drugs (A). shows a Venn diagram of positive results in four different algorithms for AEs caused by seven different drugs in FAERS database (B). shows a Venn diagram of positive results in four different algorithms for AEs caused by seven different drugs in JADER database (C). shows a Venn diagram of positive results in four different algorithms for AEs caused by seven different drugs in CVARD database

Figure S9. Heatmap Visualization of ROR Signal Intensities for the Top Ten PTs in Nervous System and Blood and Lymphatic System Disorders across FAERS, JADER, and CVARD Databases (A). The heatmap shows the ROR signal intensities of the top ten PTS of nervous system disorders SOC in FAERS database (B). The heatmap shows the ROR signal intensities of the top ten PTs of blood and lymphatic system disorders SOC in FAERS database (C). The heatmap shows the ROR signal intensities of the top ten PTS of nervous system disorders SOC in JADER database (D). The heatmap shows the ROR signal intensities of the top ten PTs of blood and lymphatic system disorders SOC in JADER database (E). The heatmap shows the ROR signal intensities of the top ten PTS of nervous system disorders SOC in CVARD database (F). The heatmap shows the ROR signal intensities of the top ten PTs of blood and lymphatic system disorders SOC in CVARD database

Figure S10. Signals detection at the HLT and HLGT level. (A).Forest plot arranged according to ROR values of HLT in blood and lymphatic system disorders SOCs in FAERS database (B).Forest plot arranged according to ROR values of HLT in nervous system disorders SOCs in FAERS database (C). Sankey plots of the top ten most numerous PTs and corresponding HLT,HLGT, SOC in blood and lymphatic system disorders SOCs in FAERS database (D). Sankey plots of the top ten most numerous PTs and corresponding HLT,HLGT, SOC in nervous system disorders SOCs in FAERS database (E).Forest plot arranged according to ROR values of HLT in blood and lymphatic system disorders SOCs in JADER database (F).Forest plot arranged according to ROR values of HLT in nervous system disorders SOCs in JADER database (G). Sankey plots of the top ten most numerous PTs and corresponding HLT,HLGT, SOC in blood and lymphatic system disorders SOCs in JADER database (H). Sankey plots of the top ten most numerous PTs and corresponding HLT,HLGT, SOC in nervous system disorders SOCs in JADER database (I).Forest plot arranged according to ROR values of HLT in blood and lymphatic system disorders SOCs in CVARD database (J).Forest plot arranged according to ROR values of HLT in nervous system disorders SOCs in CVARD database (K). Sankey plots of the top ten most numerous PTs and corresponding HLT,HLGT, SOC in blood and lymphatic system disorders SOCs in CVARD database (L). Sankey plots of the top ten most numerous PTs and corresponding HLT,HLGT, SOC in nervous system disorders SOCs in CVARD database

Figure S11.Cumulative incidence of AEs induced by GSK3β inhibitors (A).Cumulative incidence of AEs induced by seven different drugs from the time of treatment initiation to the date of occurrence (B).-(I). Cumulative incidence of AEs induced by seven different drugs from the time of treatment initiation to the date of occurrence of the nervous system disorders and blood and lymphatic system disorders SOCs

Figure S12.TTO Analysis of FAERS database. (A).-(H). shows the density of the TTO for AEs induced by seven different drugs at different time periods when it occurred (I).-(P). Two-way bar graph of the percentage and case number of TTO for AEs induced by seven different drugs in different time periods

Figure S13. Volcano plot of PTs for male and female subgroups in FAERS database. (A).Volcano plot of PTs for male and female subgroups in FAERS database ranked by log_3_(ROR) (B).Volcano plot of PTs for male and female subgroups in FAERS database ranked by log_2_(PRR)
